# Integrated multi-omics analyses on patient-derived CRC organoids highlight altered molecular pathways in colorectal cancer progression involving PTEN

**DOI:** 10.1186/s13046-021-01986-8

**Published:** 2021-06-21

**Authors:** Marta Codrich, Emiliano Dalla, Catia Mio, Giulia Antoniali, Matilde Clarissa Malfatti, Stefania Marzinotto, Mariaelena Pierobon, Elisa Baldelli, Carla Di Loreto, Giuseppe Damante, Giovanni Terrosu, Carlo Ennio Michele Pucillo, Gianluca Tell

**Affiliations:** 1 Laboratory of Molecular Biology and DNA Repair, Department of Medicine, University of Udine, Piazzale M. Kolbe 4, 33100 Udine, Italy; 2 Institute of Medical Genetics, Department of Medicine, University of Udine, 33100 Udine, Italy; 3 Piattaforma Specializzata Centro Servizi e Laboratori, Area Biologia Molecolare, Colture Cellulari e Supporto, Azienda Sanitaria Universitaria Friuli Centrale, 33100 Udine, Italy; 4 School of Systems Biology, Center for Applied Proteomics and Molecular Medicine, George Mason University, Manassas, VA 20108 USA; 5 Center for Applied Proteomics and Molecular Medicine, George Mason University, Manassas, VA 20108 USA; 6 Pathology Unit, Department of Medicine, University of Udine, 33100 Udine, Italy; 7 General Surgery and Transplantation Unit, Department of Medicine, University of Udine, 33100 Udine, Italy; 8 Laboratory of Immunology, Department of Medicine, University of Udine, 33100 Udine, Italy

**Keywords:** Organoids, Colorectal cancer, Whole exosome sequencing, RNA-seq, PTEN

## Abstract

**Background:**

Colorectal cancer (CRC) represents the fourth leading cause of cancer-related deaths. The heterogeneity of CRC identity limits the usage of cell lines to study this type of tumor because of the limited representation of multiple features of the original malignancy. Patient-derived colon organoids (PDCOs) are a promising 3D-cell model to study tumor identity for personalized medicine, although this approach still lacks detailed characterization regarding molecular stability during culturing conditions. Correlation analysis that considers genomic, transcriptomic, and proteomic data, as well as thawing, timing, and culturing conditions, is missing.

**Methods:**

Through integrated multi–omics strategies, we characterized PDCOs under different growing and timing conditions, to define their ability to recapitulate the original tumor.

**Results:**

Whole Exome Sequencing allowed detecting temporal acquisition of somatic variants, in a patient-specific manner, having deleterious effects on driver genes CRC-associated. Moreover, the targeted NGS approach confirmed that organoids faithfully recapitulated patients’ tumor tissue. Using RNA-seq experiments, we identified 5125 differentially expressed transcripts in tumor versus normal organoids at different time points, in which the PTEN pathway resulted of particular interest, as also confirmed by further phospho-proteomics analysis. Interestingly, we identified the *PTEN* c.806_817dup (NM_000314) mutation, which has never been reported previously and is predicted to be deleterious according to the American College of Medical Genetics and Genomics (ACMG) classification.

**Conclusion:**

The crosstalk of genomic, transcriptomic and phosphoproteomic data allowed to observe that PDCOs recapitulate, at the molecular level, the tumor of origin, accumulating mutations over time that potentially mimic the evolution of the patient’s tumor, underlining relevant potentialities of this 3D model.

**Supplementary Information:**

The online version contains supplementary material available at 10.1186/s13046-021-01986-8.

## Background

Colorectal cancer (CRC) is one of the most common cancers, causing around 10% of worldwide mortality and placing as the fourth leading cause of cancer-related deaths [1–3]. As with several other types of tumors, in which diet and environmental factors play primary roles in tumor generation, somatic genetic variations have a leading role in CRC initiation and/or progression [4, 5]. Several chromosomal and sub-chromosomal aberrations, as well as amplification and loss of heterozygosity, have been found in CRC [3, 6]*.* Furthermore, frequent mutations are carried on different genes such as *WNT*, *RAS/MAPK*, *PI3K*, *TGF-ß*, altering the WNT pathway and mTOR signaling, as well as occurring on tumor suppressors including *ARID1A*, *CTNNB1*, *DCC*, *FAM123B*, *FBXW7*, *PTEN*, *RET*, *SMAD4* and *TGFBR2*, and proto-oncogenes including *BRAF*, *ERBB2*, *GNAS*, *IGF2*, *KRAS*, *MYC*, *NRAS*, *RSPO2/3*, *SOX9* and *TCF7L2* [7, 8]. Interestingly, not only genetic but also epigenetic alterations, including DNA methylation and histone modifications, play a role in the initiation/progression of CRC [9]. Several CRC tumors are also characterized by microsatellite instability (MSI), as a result of the loss-of-function of crucial enzymes of the DNA mismatch repair (MMR) system (i.e. MLH1, MSH2, MSH6 and PMS2) [10]. Due to the high heterogeneity of CRC, in the last years, numerous studies have been conducted to explore novel potential biomarkers having prognostic and predictive value for this disease. In this context, genes involved in cell survival and different DNA repair pathways have also been associated with CRC susceptibility. Besides high penetrance mutations of genes in heredofamilial colon cancer, such as *APC*, *MUTYH* and those encoding proteins of the MMR, *P53* and *MDM2* families, polymorphisms could be definitely considered candidate risk factors for CRC [11]. Additionally, our recent publication demonstrated that base excision repair (BER) enzymes expression, including DNA glycosylases, APE1 and Pol ß, resulted in altered in CRC [12, 13].

Due to heterogeneous tumor identity, characterizing CRC etiology, and a missing interaction with the microenvironment, the usage of cell lines, although widely employed to study cancer biology, has limited the research of CRC over time. Recent advances in stem cell culture have allowed us to derive in vitro tridimensional cultures called organoids able to recapitulate anatomical and functional hallmarks of the real organ, boosting our understanding of the mechanisms of tumorigenesis and malignancy progression [14].

Organoids represent a novel in vitro technology, which allows the establishment of long-term stem cell-based three-dimensional (3D) cultures. Organoids can be generated from tissue-resident adult stem cells capable of self-organizing in tissue-specific cell types, recreating the typical microanatomy of the native organs [15]. Although organoids have been generated from many different organs, those derived from the gastrointestinal tract including: small intestine, colon, stomach, esophagus, liver and pancreas are the most studied [16]. Since the first establishment of the organoid protocol to enable culturing of the small intestine from the whole crypts and a single Lgr5^+^ stem in 2009 by the Clevers’ group, several works have further emphasized the role of CRC organoids as a powerful tool in basic and translational research [15, 17]. After these achievements, multiple collections of CRC organoids derived from clinical biopsies of patients have been characterized [16]. In particular, accumulating evidence indicated that patient-derived colon organoids (PDCOs) displayed similar genomic and transcriptomic profiles as their original tumors and, more interestingly, can be used to perform high-throughput drug screening for predicting treatment response [18–24]. Given the importance of clinical application of PDCOs in drug discovery, therapy response and personalized medicine, we here focused on colon organoids derived from patients affected by CRC. As aforementioned, several studies involving PDCOs have been already conducted; however, even if a correlation analysis of genomic, transcriptomic and proteomic data already exists [18, 25], a methodic study taking simultaneously into account all these aspects, which combines different timing and culturing conditions as well as considers the variable due to the thawing process, is actually lacking [26]. Here, we provided a complete characterization of PDCOs’ features under different culturing environments through integrated multi–omics strategies.

This study aims to characterize, at the molecular level and by using different technologies, the altered molecular pathways in CRC progression and the stability of PDCOs in culture.

## Methods

### Human tissues and organoid culture

Colon tumor and adjacent normal tissues were obtained upon surgical resection from three patients (identified as: P12, P14 and P16) from the University Hospital “Santa Maria della Misericordia” of Udine. The size of the surgically resected intestinal tissues was on average 0.5 cm. A distance of at least 3 cm from the tumor was maintained for normal tissue collection. Normal and tumor tissues were processed immediately after resection. P14 and P16 were diagnosed with adenocarcinoma whereas P12 with mucinous adenocarcinoma. This study was approved by the ethical committee of the University Hospital “Santa Maria della Misericordia” of Udine (CEUR-2017-PR-048-UNIUD) and informed consent was obtained before sample collection.

The generation of patient-derived organoids was performed as described by [18, 27]. For further details see Supplementary Data.

### Immunohistochemistry

Organoids were collected in a cold basal medium and Matrigel was mechanically dissociated. After the fixation in paraformaldehyde (4%) for 20 min at RT, organoids were included in agarose solution (1%). Organoids in agarose solution were paraffin-embedded following dehydration in ascending series of alcohol and clarification in xylene. From paraffin blocks, we cut 5 μm multiple serial sections on a microtome. The first of each series of 5 sections was stained with hematoxylin and eosin by routine method and read under a light microscope to judge the morphology and to choose the best levels for immunohistochemical stains. The VENTANA MMR immunohistochemistry Panel was used for the assessment of mismatch repair (MMR) proteins (MLH1, PMS2, MSH2, and MSH6) in colorectal cancer and organoids tissue sections on the VENTANA BenchMark ULTRA instrument. The VENTANA MMR Panel includes the anti-MLH1 (M1), anti-PMS2 (A16–4), anti-MSH2 (G219–1129) mouse monoclonal primary antibodies and the anti-MSH6 (SP93) rabbit monoclonal primary antibody, all ready to use. The binding of the primary antibody to MLH1, MSH2, MSH6 was detected by the OptiView DAB IHC Detection Kit, while the OptiView DAB IHC Detection Kit with the OptiView Amplification Kit were used for PMS2. All slides were processed according to the manufacturer’s protocol with proprietary reagents using Cell Conditioning 1 (pH 9.0) and heat-induced antigen retrieval. Incubation time varied with each biomarker. In each staining run, one section was stained with a negative reagent control antibody. Nuclei of normal and neoplastic cells labelled with the immunohistochemical assays for four MMR proteins (MLH1, PMS2, MSH2 and MSH6) were evaluated for the presence or loss of staining signals. Positive nuclear staining indicated an intact MMR status, whereas the absence of detectable nuclear signal, in the presence of appropriately stained internal controls, indicated a loss of MMR protein expression.

### Microsatellite instability (MSI) evaluation

Microsatellite Instability (MSI) was evaluated using an AlphaCapillary MSI Kit (Alphagenics Biotech). The multiplex microsatellite panel comprises 12 markers: 5 Mono-nucleotides (TGFBR2, BAT26, BAT25, MT1XT20 and BAT40), 4 of Di-nucleotide (D2S123, D5S346, D17S250 and D18S58) and 3 of Tetra-nucleotides (D7S820, D18S51 and CSF1PO). Microsatellite loci were amplified using a fluorescent PCR-based assay. In detail, fluorescently labeled oligonucleotides conjugate with HEX or FAM and dyes were used in two multiplex PCR containing of each primer (20 μM), dNTP (200 μM), MgCl_2_ (1.5 mM) and Taq DNA polymerase (0.75 units). DNA (10 ng) was used as a template for each reaction. The amplification protocol was carried out using the following conditions: denaturation at 95 °C for 15 min, 35 cycles of denaturation at 94 °C for 30 s, annealing at 57 °C for 90 s and extension at 72 °C for 60 s, followed by an extension step for 72 °C for 10 min.

The amplification products were analyzed using an automated capillary electrophoresis system (ABI PRISM 3500 DX) in order to discriminate allelic length, using the GENEMAPPER 5.0 software.

### Whole exome sequencing (WES)

The SureSelectXT Target Enrichment System for Illumina Paired-End Multiplexed Sequencing Library protocol was used to generate libraries that were sequenced using NextSeq 500 (Illumina, San Diego, CA) (151 bp read length). Raw reads quality was evaluated using the ShortRead (v1.44.3) R/Bioconductor package [28]. Somatic variants were called via local assembly of haplotypes following the GATK somatic short mutation calling best practice workflow (https://gatk.broadinstitute.org/hc/en-us/articles/360035531132. For further details see Supplementary Data.

### Library preparation and targeted NGS

Genomic DNA was extracted using the QIAamp DNA Mini Kit (Qiagen) and quantified using the Qubit dsDNA HS Assay Kit (Life Technologies). Barcoded libraries were generated, as previously described in [29]. Annotation was performed with both Ion Reporter 5.10 (Thermo Fisher Scientific) and wANNOVAR [30]. Somatic variants were called when a position was covered at least 500X. We set the clinical sensitivity of point mutations and indels at 5%. Variant prioritization was based on the pipeline described in [12] .

### Sanger sequencing

Amplification was performed using 20 ng of PDCO-extracted DNA and Platinum™ II Hot-Start PCR master mix (Thermo Fisher Scientific). PCR primer sequences are available on demand. The amplified products were analyzed by direct sequencing using the Big Dye Terminator Cycle Sequencing Kit v3.1 and capillary electrophoresis on the 3500 Dx Series Genetic Analyzer (Applied Biosystems).

### RNA sequencing and data analysis

Sequencing of TruSeq Stranded mRNA samples was carried out on paired-end 101 bp mode on NextSeq 500 (Illumina, San Diego, CA). Sequencing reads quality evaluation, filtering, alignment, transcript assembly, quantification, differentially expressed genes identification and downstream analysis were performed using R/Bioconductor packages in R (versions 3.6.1 and 3.6.2) using RStudio (v1.2). For further details see Supplementary Data.

### Reverse phase protein microarray

Organoids were lysed on ice in TPER Reagent (Thermo Fisher), supplemented with NaCl (300 mM) and a cocktail of protease and phosphatase inhibitors as previously described [31]. Protein concentrations were assessed using the Coomassie (Bradford) Protein Assay Kit (Thermo Fisher Scientific Waltham, MA) following the manufacturer’s instructions. Samples were then diluted in SDS-PAGE 4X Sample Buffer supplemented with β-mercaptoethanol (10%) to a final concentration of 0.5 mg/mL, boiled for 8 min, and stored at − 80 °C until arrayed.

Cell lysates were immobilized onto nitrocellulose coated glass slides (Grace Bio-labs, Bend, OR) using an Aushon 2470 arrayer (Aushon BioSystems, Billerica, MA) equipped with 185 μm pins. Each sample was printed in technical replicates (*n* = 3) along with reference standard curves used for internal QA/QC [32]. For normalization purposes, the protein amount for each sample was assessed on selected arrays using a Sypro Ruby Protein Blot Stain (Molecular Probes, Eugene, OR) protocol following the manufacturer’s instructions. Before undergoing immunostaining, the remaining arrays were treated with Reblot Antibody Stripping solution (Chemicon) and incubated for at least 1 h with I-block (Tropix). Arrays were probed with 120 monoclonal or polyclonal antibodies, of which 103 targeted post-translational modified proteins. Antibodies specificity for their target analyte was assessed by Western Blotting as previously described [33]. Primary antibodies were recognized by a biotinylated anti-rabbit or anti-mouse secondary antibody (Vector Laboratories, Inc.). Selected arrays were incubated with the secondary antibodies alone to capture background and unspecific signals. Signal detection was achieved using a commercially available tyramide-based avidin/biotin amplification system (Genpoint Kit; Dako Cytomation) coupled with IRDye 680RD Streptavidin (LI-COR Biosciences) according to the manufacturer’s instruction [34, 35]. Antibody and Sypro Ruby stained slides were scanned on a laser scanner (TECAN). Images were analyzed using the MicroVigene Software Version 5.1.0.0 (Vigenetech) as previously described [34, 35]. In brief, samples were normalized to their corresponding total protein values (Sypro Ruby stained slides). Background and unspecific signals were then subtracted, and technical replicates (*n* = 3) were averaged to generate single-values for each experimental sample on a continuous scale. To explore changes in the phosphoprotein-based signaling profiles, unsupervised hierarchical clustering analysis was performed using the Ward method in JMP software 5.1.2 (SAS Institute Inc., Cary, NC, USA).

## Results

### Establishment of patient-derived colon organoids (PDCOs) 3D-cell cultures

**Fig. 1:**
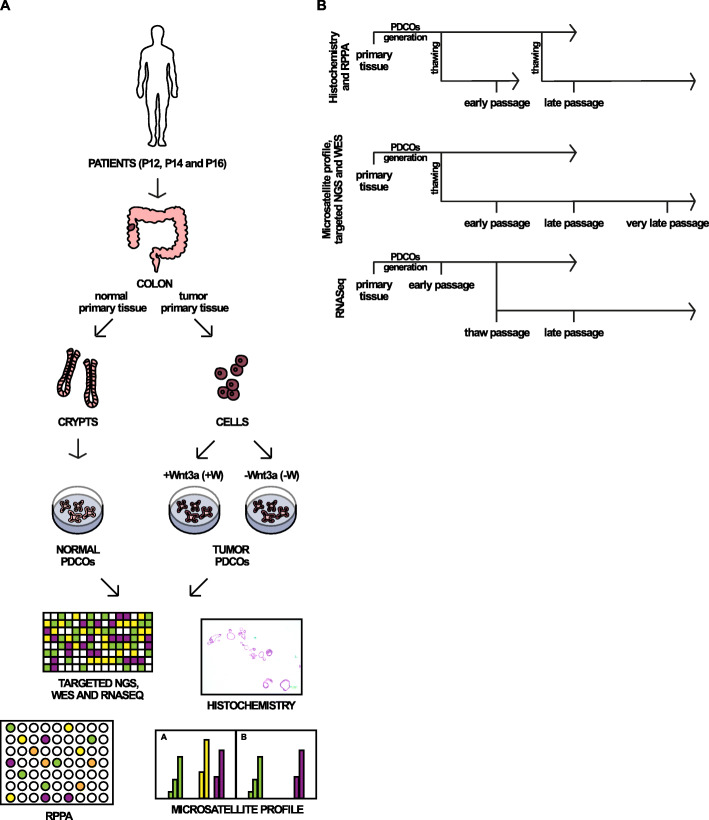
Schematic representation of the experimental workflow. **A** Patient-derived colon organoids (PDCOs) were generated from 3 patients affected by CRC. The crypts derived from normal tissue and the isolated cells from tumor were used to generate normal and tumor PDCOs, respectively. Normal PDCOs were grown in medium added with the Wnt3a factor (+W), whereas tumor PDCOs in medium in the presence (+W) or absence of the Wnt3a factor (−W). PDCOs’ genomic DNA (gDNA), RNA and protein were collected and were analyzed by whole-exome sequencing (WES), microsatellite profile, Targeted NGS, RNA-seq, reverse-phase protein microarrays (RPPA) and histochemistry. **B** A scheme of the experimental settings for each type of analysis is indicated

PDCOs were generated from surgically resected tumor and normal intestinal mucosa obtained from three patients affected by colon cancer (P12, P14 and P16) (Supplementary Table 1 and Fig. 1). The crypts, derived from normal tissue, and the isolated cells, derived from the tumor, were considered primary tissues. From each primary tissue, we generated normal (P12N, P14N, P16N) and tumor (P12T, P14T, P16T) PDCOs. Representative images of the morphology of the PDCOs are shown in Supplementary Fig. 1. Hematoxylin and eosin staining of tumor tissue and PDCOs of P12 and P14 are shown in Supplementary Fig. 2A. PDCOs were positive for the most common intestinal markers, such as Ki67, OLFM4, E-Cadherin and Lysozyme [12]. The experimental set-up plan is described in Fig. 1. Briefly, normal PDCOs were grown in medium added with the Wnt3a factor (+W), whereas tumor PDCOs in medium in the presence or absence of the Wnt3a factor (+W or -W, respectively). Since *a priori* we did not know which mutations the PDCOs harbored, we decided to culture organoids in both +W and -W conditions. Indeed, in normal intestinal organoids the Wnt3a factor is essential for survival and growth [36]. Disruption of APC drives activation of the WNT signaling pathway, culminating in a transcriptional response [37]. Not all tumors, however, carry mutations in the WNT pathway genes and therefore the culturing of these tumor organoids is Wnt factor-dependent [27]. Therefore, regarding our tumor organoids, the culturing conditions in the presence or absence of Wnt were tested to check the dependency on this factor. PDCOs’ genomic DNA (gDNA), RNA and proteins were collected at early (passages 4–7), late (passages 11–13) and very late (passage 17) timing from the generation. Moreover, we included a thawing stage (passage 9) (Fig. 1B). Primary tissues and PDCOs were analyzed by histochemistry, microsatellite profile, whole-exome sequencing (WES), targeted NGS, RNA-seq, and reverse-phase protein microarrays (RPPA). An overview of the collected samples with the corresponding type of performed analysis is shown in Supplementary Table 2.

### Characterization of microsatellite profile of PDCOs

Microsatellite profile of tumor tissues and PDCOs of patients P12, P14 and P16 was firstly evaluated by immunohistochemistry for the MLH1, MSH2, MSH6 and PMS2 proteins. The expression of these proteins was present in all conditions (Supplementary Fig. 2B and Table 1).

**Table 1:** Immunohistochemical staining for the MLH1, MSH2, MSH6 and PMS2 proteins of patients P12, P14 and P16

Patient	MLH1		MSH2		MSH6		PMS2	
	Tissue	PDCO (% positive)	Tissue	PDCO (% positive)	Tissue	PDCO (% positive)	Tissue	PDCO (% positive)
P12	MSS	95	MSS	100	MSS	95	MSS	100
P14	MSS	90	MSS	100	MSS	100	MSS	70
P16	MSS	80	MSS	100	MSS	100	MSS	70

Microsatellite analysis obtained for each CRC tissue section and paired PDCOs is indicated in the table. The percentage of positive cells for the MLH1, MSH2, MSH6 and PMS2 proteins in tumor PDCOs relative to total cells is represented

To analyze microsatellite status, the P12 and P14 patients were evaluated by PCR and capillary electrophoresis using a multiplex microsatellite panel. In this analysis, we included normal and tumor primary tissues, as well as PDCOs, collected following different timing and growth conditions (Fig. 1B). The MSI spectrum of the 12 tested molecular markers revealed that the microsatellite profile was conserved between tumor and matched normal counterparts in the P12 patient. Furthermore, PDCOs of P12 did not show major differences in microsatellite repeats among different culture conditions (+W versus -W) and over time (Table 2).

**Table 2:** Characterization of the PDCOs microsatellite profile by PCR and capillary electrophoresis using a multiplex microsatellite panel

P	N/T	Sample	M	Pass	Marker											
					BAT26	D17S250	TGFBR2	D2S123	D5S3346	CSF1PO	BAT25	D18S58	Mt1xT20	D7S820	BAT40	D18S51
**P12**	T	primary tissue	/	0	MSS	MSS	MSS	MSS	MSS	MSS	MSS	MSS	MSS	MSS	MSS	MSS
	N	PDCO	+W	early	MSS	MSS	MSS	MSS	MSS	MSS	MSS	MSS	MSS	MSS	MSS	MSS
	N	PDCO	+W	late	MSS	MSS	MSS	MSS	MSS	MSS	MSS	MSS	MSS	MSS	MSS	MSS
	T	PDCO	+W	ealy	MSS	MSS	MSS	MSS	MSS	MSS	MSS	MSS	MSS	MSS	MSS	MSS
	T	PDCO	-W	early	MSS	MSS	MSS	MSS	MSS	MSS	MSS	MSS	MSS	MSS	MSS	MSS
	T	PDCO	-W	late	MSS	MSS	MSS	MSS	MSS	MSS	MSS	MSS	MSS	MSS	MSS	MSS
**P14**	T	primary tissue	/	0	MSS	MSS	MSS	MSS	MSS	MSS	MSS	MSS	MSS	MSS	MSS	MSS
	N	PDCO	+W	early	MSS	MSS	MSS	MSS	MSS	MSS	MSS	MSS	MSS	MSS	MSS	MSS
	T	PDCO	+W	early	MSS	MSS	MSS	MSS	MSI	MSI	MSS	MSS	MSS	MSS	MSS	MSI
	T	PDCO	+W	late	MSS	MSS	MSS	MSS	MSI	MSI	MSS	MSS	MSS	MSS	MSS	MSI
	T	PDCO	+W	very late	MSS	MSS	MSS	MSS	MSI	MSI	MSS	MSS	MSS	MSS	MSS	MSI
	T	PDCO	-W	early	MSS	MSS	MSS	MSS	MSI	MSI	MSS	MSS	MSS	MSS	MSS	MSI
	T	PDCO	-W	late	MSS	MSS	MSS	MSS	MSI	MSI	MSS	MSS	MSS	MSS	MSS	MSI

*P* Patient, *M* Medium, *Pass* Passage

Contrarily to P12, most of the samples recovered from P14 displayed a loss of heterozygosity (LOH) of different genomic markers, a typical cancer molecular signature. Specifically, tumor PDCOs from P14 harbored a monoallelic clonal loss of the D5S346, CSF1PO and D18S51 markers, a typical microsatellite instability (MSI) phenotype. The complete electropherograms are shown in Supplementary Fig. 3. This switch from MSS to MSI described in P14, was independent of culture conditions (+W versus -W) and timing (Table 2). In conclusion, these data suggest that tumor organoids partially recapitulate the microsatellite profiles of the primary tissue.

### Genomic characterization of PDCOs through WES analysis

We used Mutect2 to analyze paired tumor-normal exomes to identify tumor-specific single nucleotide variants (SNVs) or insertions and deletions (indels) that could be involved in the oncogenic process. Specifically, we first compared normal and tumor primary tissues and, afterwards, normal and tumor PDCOs; then, we evaluated the acquisition of variants by the organoids lacking in the primary tissues (both tumor and normal component). Finally, we assessed if growth media or different culture times may affect PDCOs genomic instability. Altogether, we performed twenty-five comparisons (Table 3) covering the most interesting changes in patient, time and experimental conditions, identifying hundreds of SNVs and indels in each iteration (*n* = 472 on average). As expected, we identified fewer variants in closely related samples (e.g., the same sample profiled at two consecutive time points, or primary tissue and associated PDCOs). The number of alterations increased with the increase of biological/experimental differences between the examined samples and reached the top in “normal primary tissue *versus* late-time tumor PDCO” comparisons. We observed a significant difference (*p* = 0.00078) in the average number of called variants between patient P12 (*n* = 249) and patient P14 (*n* = 600), comparing the eight common experimental conditions (“N early_N tissue”, “T -W early_N early”, “T -W early_T tissue”, “T tissue_N early”, “T tissue_N tissue”, “T +W early_N early”, “T +W early_T -W early” and “T +W early_T tissue”). This information corroborated our data about microsatellite stability, confirming previous observations, which pointed to sample P14 as characterized by higher genomic instability. On the contrary, we found no difference in deleterious variants percentages defined by SIFT and CLINVAR and in non-synonymous ones, with an average of 22, 1 and 77%, respectively.

**Table 3:** Summary of WES deleterious and potentially deleterious somatic variants. We performed twenty-five comparisons, identifying hundreds of SNVs and indels in each iteration (*n* = 472 on average)

Comparison	# Variants	Type			Localization				
		SIFT	ClinVar	Non-Synonymous	Exonic	ncRNA_exonic	Splicing	5’UTR	3’UTR
P12T primary tissue_P12N primary tissue	532	108	7	417	141	51	5	50	285
P12N early_P12N primary tissue	248	56	1	191	80	30	4	26	108
P12N late_P12N early	139	24	1	114	37	20	1	18	63
P12T primary tissue_P12N early	506	103	7	396	135	47	5	45	274
P12T + W early_P12N early	328	93	7	228	114	30	2	38	144
P12T -W early_P12N early	435	113	9	313	150	41	6	58	180
P12T -W late_P12N late	313	82	7	224	107	20	2	33	151
P12T -W late_P12T -W early	155	22	0	133	30	19	0	23	83
P12T + W early_P12T primary tissue	153	30	1	122	39	24	0	25	65
P12T -W early_P12T primary tissue	223	38	2	183	54	31	5	43	90
P12T + W early_P12T -W early	152	26	0	127	33	26	0	23	71
P14T primary tissue_P14N primary tissue	592	90	3	499	134	49	6	72	331
P14N early_P14N primary tissue	257	60	2	195	85	26	1	38	107
P14T primary tissue_P14N early	661	95	3	563	142	61	6	77	375
P14T + W early_P14N early	815	219	8	588	265	71	11	122	346
P14T + W late_P14N early	728	192	10	526	238	69	8	107	306
P14T + W very late_P14N early	747	203	9	535	255	69	7	110	306
P14T -W early_P14N early	789	213	10	566	261	66	9	98	355
P14T -W late_P14N early	858	234	11	613	296	86	9	106	361
P14T + W early_P14T primary tissue	604	150	7	447	188	55	9	84	268
P14T -W early_P14T primary tissue	585	142	8	435	183	61	8	66	267
P14T + W early_P14T -W early	301	58	0	243	74	34	5	56	132
P14T + W late_P14T primary tissue	518	123	8	387	160	47	6	74	231
P14T -W late_P14T primary tissue	712	179	10	523	240	71	8	77	316
P14T + W late_P14T -W late	450	101	6	343	134	47	7	74	188

During the variant calling analyses, we focused on variants mapping to exons (coding and non-coding), splice junctions and UTRs. The majority of the identified variants (46%) fall within 3’UTRs, suggesting the deregulation of tumor-related events such as: mRNA processing, stability, localization, polyadenylation and miRNA binding. Coding exons harbor 29% of variants, followed by 5’UTRs and ncRNA exons with 13 and 11%, respectively, with splice junctions associated with the remaining 1%. We did not find significant differences between patients P12 and P14 overall variants location; however, patient P12 showed a slightly higher percentage of variants associated with ncRNA exons than patient P14 (11 and 9.6%, respectively; *p* = 0.046).

**Fig. 2:**
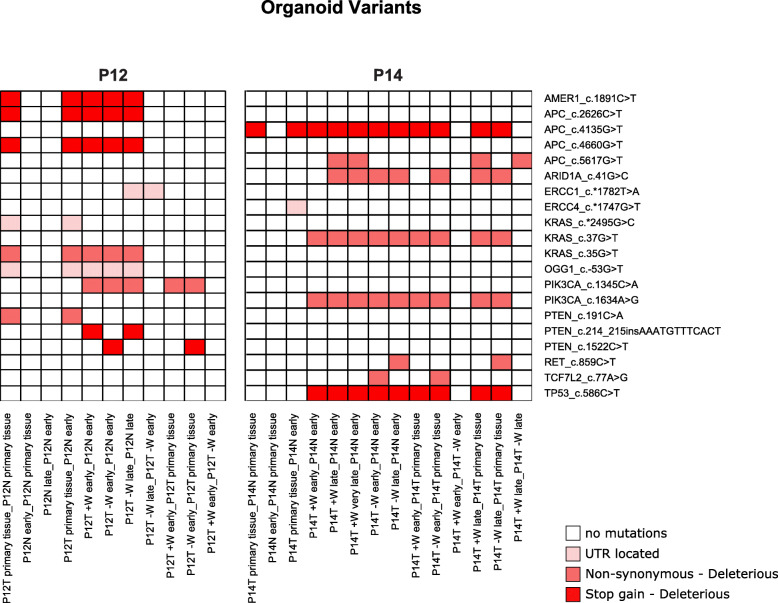
Somatic variants in colorectal cancer driving genes. Heatmap summarizing the somatic mutations identified in twelve genes associated with CRC and DNA-damage response pathways. Results originated from twenty-five comparisons that were performed between tumor/normal PDCOs and/or their primary tissues and are split based on patient. The severity of variants is color-coded

Finally, we focused on a subset of forty-seven genes associated with CRC and DNA-damage response (DDR) pathways, identifying variants in twelve of these genes (Fig. 2) [3, 38, 39]. *APC*, *PIK3CA* and *KRAS* were the genes most affected by genomic instability, acquiring (in primary tumor versus normal tissue) or undergoing an increase of the allele frequencies (AF) (in PDCOs vs primary tumor) of stop codons or non-synonymous, deleterious variants in 17 (*APC*) and 14 (*PIK3CA* and *KRAS*) of the experimental conditions. In particular, *APC* was the most affected gene and 3 of the identified variants lead to a stop codon gain. In patient P14, *TP53* displayed a stop gain variant conserved in several experimental conditions. A similar trend was also detected for *AMER1* in patient P12. The remaining DDR genes acquired deleterious mutations or variants mapping to UTRs (*ERCC1*, *ERCC4* and *OGG1*).

Examining the distribution of variants in the twelve DDR genes, normal primary tissues and PDCOs are devoid of any variants, indicating that the procedure we used for organoids generation does not lead to artifactual variants. Patient P14 PDCOs seem to represent an earlier stage of tumorigenesis, following the IHC analysis, since the primary tumor acquired only one variant (*APC*_chr5_112839729_112839729_G_T) with respect to its normal primary tissue. Confirming this, the “P14T primary tissue_P14N early” comparison is associated with a lower number of variants (*n* = 661), if compared to other P14 conditions, and it has the lowest percentage of predicted SIFT deleterious variants (14%) among all the profiled conditions. This could be also related to P14T PDCOs showing a higher number of variants at later time points, possibly due to delayed oncogenic events. Variants seem to follow a patient-specific, modular pattern. Those affecting the same gene (e.g., *APC*, *KRAS* and *PIK3CA*), tend to be mutually exclusive. We observed two groups of co-occurring variants in the same conditions in patient P12 (*AMER1*_chrX_64191396_64191396_G_A, *APC*_chr5_112838220_112838220_C_T, *APC*_chr5_112840254_112840254_G_T, *KRAS*_chr12_25245350_25245350_C_A, *OGG1*_chr3_9750234_9750234_G_T) and in patient P14 (*APC*_chr5_112839729_112839729_G_T, *KRAS*_chr12_25245348_25245348_C_A, *PIK3CA*_chr3_179218304_179218304_A_G, *TP53*_chr17_7674945_7674945_G_A), suggesting these genes could operate in a coordinated manner during carcinogenesis. Growth timing and media seem to have a mild effect on variants acquisition by PDCOs. Regarding growth time, we observed the appearance of a UTR variant in the *ERCC1* gene in the “P12T -W late_P12T -W early” comparison. Moreover, we observed a deleterious, non-synonymous variant in the P14T + W late and P14T + W very late samples compared to early P14N, missing in P14T + W early PDCOs. Based on this evidence, PDCOs at earlier stages already recapitulate the majority of later events. Culture time played a more important role in P14 PDCOs since most of the variants were missing in the primary tumor tissue and were identified only from the early stages of PDCOs onwards. Considering growth medium conditions, in a couple of comparisons (“P12T -W early_P12T primary tissue” and “P14T -W late_P14T primary tissue”) we observed variants in the *PTEN* and *RET* genes that were not present in the corresponding T + W early sample; however, when directly comparing T -W early and T + W early samples, these variants disappeared indicating similar AF. The only permanent medium effect was observed in the *APC* gene, where a deleterious variant was acquired in the P14T + W late sample compared to P14T -W late.

We already described most of the PDCOs-specific deleterious variants in P14 PDCOs (*TP53*, *PIK3CA* and *KRAS*) but this specificity, to a lesser extent, also involves sample P12. Indeed, *PIK3CA* and *PTEN* genes harbor variants that are present in samples P12T + W early and P12T -W early, but not in the P12T primary tissue; however, variants are not identified when directly comparing PDCOs to each other, suggesting an AF not significantly higher than in primary tumors.

### Targeted NGS profiling of PDCOs

CRC may arise from one or a combination of different mechanisms, the leading ones being represented by chromosomal instability (CIN), the CpG island methylator phenotype (CIMP) and MSI [3, 38, 40]. Therefore, we created an *in-house* NGS panel focused on CRC- and DNA repair-related genes, exploiting greater sequencing coverage to better characterize variants identified by WES (i.e., in terms of AF) and to identify new variants undetected by WES due to lower resolution. We used Ion Reporter and wANNOVAR to analyze paired tumor-normal samples and identify SNVs and/or indels in the selected forty-five genes. We selected P12 and P14 normal and tumor primary tissues together with the relative PDCOs, grown in -W medium at early passages. We set the clinical sensitivity of point mutations and indels at 5% and we excluded synonymous variants from further analysis. After filtering, normal primary tissues and the derived PDCOs did not show any putative-damaging SNV. As for the tumor counterpart, tissue derived from P12 harbored a *PIK3CA* missense mutation (chr3_178928067_C_A), two *APC* stop-gain mutations (chr5_112173917_C_T; chr5_112175951_ G_T), a *PTEN* stop-gain mutation (chr10_8972066_8972078dup), and a *KRAS* missense mutation (chr12:_25398284_C_A). The PDCO from P12T -W early*,* harbored the same SNVs found in the tissue of origin, but it also acquired an additional *PTEN* stop-gain mutation (chr10_89720852_C_T).

Tumor primary tissue from P14 harbored a missense mutation of *PIK3CA* (chr3_ 178936092_A_G), an *APC* stop-gain mutation (chr5_112175426_G_T), a *KRAS* missense mutation (chr12_25398282_C_A) and a *TP53* stop-gain mutation (chr17_7578263_C_T). The PDCO from P14 harbored the same mutations found in the tissue of origin. Table 4 summarizes the genetic alterations assessed in PDCOs and tissues.

**Table 4:** Genetic alterations assessed through targeted NGS profiling in CRC primary tissues and relative PDCOs

Sample ID	Gene ID	Protein Change	VAF (%)	COSMIC Classification
**P12T** **primary tissue**	PIK3CA	P449T	16	Pathogenic
	APC	R876X	34	Pathogenic
	APC	E1554X	28	Pathogenic
	PTEN	F273X	7	Unknown
	KRAS	G12V	26	Pathogenic
**P12T** **-W early**	PIK3CA	P449T	38	Pathogenic
	APC	R876X	50	Pathogenic
	APC	E1554X	50	Pathogenic
	PTEN	F273X	58	Unknown
	PTEN	R335X	20	Pathogenic
	KRAS	G12V	50	Pathogenic
**P14T** **primary tissue**	PIK3CA	E545G	12	Pathogenic
	APC	E1379X	22	Pathogenic
	KRAS	G13C	12	Pathogenic
	TP53	R196X	18	Pathogenic
**P14T** **-W early**	PIK3CA	E545G	50	Pathogenic
	APC	E1379X	100	Pathogenic
	KRAS	G13C	50	Pathogenic
	TP53	R196X	100	Pathogenic

For every variant, we indicate the variant allele fraction (VAF) and the putative pathogenicity

Indeed, most of the genetic variants detected in these samples are well-known somatic variants, reported as pathogenic in the COSMIC database (https://cancer.sanger.ac.uk/cosmic) with the exception of *PTEN* F273X. This is a 12-bases exon 8 *PTEN* duplication (NM_000314), which, to our knowledge, has never been reported before in colon cancer. This variant has been previously defined as deleterious by the American College of Medical Genetics and Genomics (ACMG)/Association for Molecular Pathology (AMP) classification (PVS1, PM1, PM2 and PP3) in the Varsome database (https://varsome.com/). This alteration was also identified by our WES analysis, but it was discarded during the filtering phase due to the lower coverage.

*PTEN* is a tumor suppressor gene, member of a eukaryotic family of lipid phosphatases named Tensin, that is involved in the inhibition of cell cycle progression, induction of cell death, modulation of arrest signal, and stimulation of angiogenesis [41]. The aforementioned 12-bp duplication creates a stop codon altering the sequence of the C-terminal C2 domain, fundamental for lipid-membrane binding, with subsequent destabilization of the entire protein and a putative alteration in its catalytic activities [42]. The c.806_817dup was confirmed by Sanger sequencing (Supplementary Fig. 4).

### Transcriptomic characterization of PDCOs through RNA-seq

**Fig. 3:**
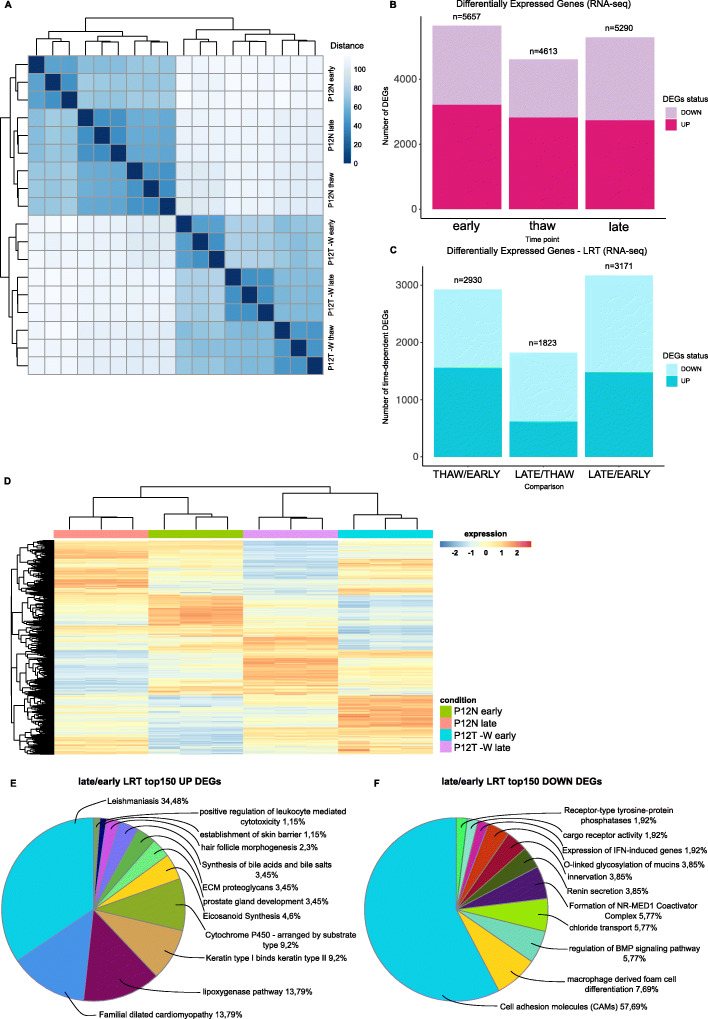
Transcriptomic profiling of normal and tumor PDCOs. **A** Sample-to-sample distance heatmap of the RNA-seq data. The vst-transformed counts matrix was used to calculate the Euclidean distance between samples; hierarchical clustering was performed using the complete agglomeration method. Samples are grouped in two main clusters, representing normal PDCOs (top left) and tumor PDCOs derived from patient P12 (−W medium; bottom right). **B** Barplot showing the differentially expressed genes (DEGs; abs(log2FC) ≥ 1, FDR < 0.05) identified by comparing tumor PDCOs derived from patient P12 (−W medium) versus normal PDCOs. Early PDCOs DEGs: *n* = 5657 (3224 up-regulated and 2433 down-regulated); thaw PDCOs DEGs: *n* = 4613 (2833 up- and 1780 down-regulated); late PDCOs DEGs: *n* = 5290 (2746 up- and 2544 down-regulated). **C** Barplot of DEGs (abs(log2FC) ≥ 1, FDR < 0.05) identified applying the likelihood ratio test (LRT) to evaluate the combined effect of condition and time progression on tumor PDCOs derived from patient P12 (−W medium) versus normal PDCOs. Thaw/early PDCOs DEGs: *n* = 2930 (1562 up-regulated and 1368 down-regulated); late/thaw PDCOs DEGs: *n* = 1823 (619 up- and 1204 down-regulated); late/early PDCOs DEGs: *n* = 3171 (1483 up- and 1688 down-regulated). **D** Heatmap showing DEGs applying the LRT to evaluate the combined effect of condition and time progression on normal and tumor PDCOs gene expression. Globally, 3171 transcripts were differentially expressed in tumor PDCOs at the late time point (1483 up-regulated and 1688 down-regulated; abs(log2FC) ≥ 1, FDR < 0.05). Hierarchical clustering of transcripts and samples using the Euclidean distance and the complete agglomeration method; expression data was vst-transformed, scaled and centered. **E**, **F** The top150 significantly up- (E) and down-regulated (F) coding genes identified in the LRT analysis were annotated using the Cytoscape plugin ClueGO. Functionally enriched terms (Benjamini-Hochberg adjusted *p* ≤ 0.05) were identified querying the CLINVAR_Human-diseases, WikiPathways, KEGG, REACTOME_Reactions, REACTOME_Pathways, GO_ImmuneSystemProcess, GO_BiologicalProcess and CORUM_CORUM-FunCat-MIPS databases. Pie chart colors match the enriched functional clusters; the most significant terms were used as cluster representatives and identifiers

To characterize the effect of different culturing conditions on gene expression, we performed RNA-seq at different time points (hereafter called “early”, “thaw” and “late”, Fig. 1B) to define the transcriptomes of tumor PDCOs derived from patient P12 and grown in -W medium, comparing them to normal PDCOs. We calculated sample-to-sample Euclidean distances, (Fig. 3A), finding two well-separated ‘normal *versus* tumors’ sample clusters, with replicates forming smaller clusters based on the timing from their generation. We confirmed this result by performing Principal Components Analysis (Supplementary Fig. 5A). Early tumor PDCOs displayed 5657 differentially expressed genes (DEGs) (Fig. 3B and Supplementary Fig. 5B), compared to the corresponding normal PDCOs, with 3224 up-regulated and 2433 down-regulated genes (abs(logFC) ≥ 1, padj≤0.05). Thaw tumor PDCOs had 4613 DEGs (2833 up- and 1780 down-regulated) (Fig. 3B and Supplementary Fig. 5C) and late tumor PDCOs had 5290 DEGs (2746 up- and 2544 down-regulated) (Fig. 3B and Supplementary Fig. 5D). Then, we applied the likelihood ratio test (LRT) to identify condition-specific differences over time. We found 2930 DEGs in the thaw/early comparison (1562 up- and 1368 down-regulated) (Fig. 3C), 1823 DEGs in the late/thaw comparison (619 up- and 1204 down-regulated) and 3171 DEGs in the late/early comparison (1483 up- and 1688 down-regulated) (Fig. 3D and Supplementary Fig. 5E-G), totally accounting for 5125 unique genes differentially expressed in a tumor specific, time-dependent manner in at least one comparison.

We then performed functional enrichment analysis on the top DEGs, focusing on the 150 most up- or down-regulated protein-coding genes identified in each tumor versus normal PDCOs comparison. At the “early” time point, enriched terms associated with the regulation and the action of ncRNAs (including a role in WNT signaling in hepatocellular carcinoma), embryonic/system development and NCAM1-mediated interactions in up-regulated genes, while down-regulated genes were associated with the action of the KRAB ZNF/KAP1 Corepressor Complex and with the CRC-related Tn antigen (Supplementary Fig. 6A-B). For “thaw”, up-regulated genes associated with angiogenesis, neurogenesis (possibly induced by a fraction of cancer stem cells), inflammation and redox activity. On the contrary, down-regulated genes partly recapitulated the functions identified for “early” down-regulated genes and with GPI-related membrane events and steroid metabolism (Supplementary Fig. 6C-D) [43]. Upregulation of genes involved in neurogenesis and inflammation (emphasizing chemokines and IL-12), epithelium morphogenesis and, interestingly, Cytochrome P450 activity were detected in the “late” samples. These genes have a well-established role in carcinogenesis. Down-regulated gene functions were similar to those observed in “early” and “thaw” (Supplementary Fig. 6E-F) [44].

Overall, DEGs profiling suggests that tumor PDCOs phenotypes are acquired over time, mainly through up-regulation. We confirmed this observation by comparing the three top 150 up-regulated gene lists to each other, identifying eleven functional terms enriched in a time-specific way. Repeating the analysis on down-regulated genes identified six terms (data not shown); however, the majority of enriched terms were unspecific. We finally investigated the tumor-specific functions that PDCOs acquired and lose over time, from the “early” to the “late” time point, comparing up- and down-regulated genes from the LRT approach (Fig. 3E and F). Up-regulated genes associated with “Inflammatory bowel disease”; “lipoxygenase and eicosanoids synthesis”, known mechanisms of CRC carcinogenesis; “functionality of keratins”, general markers of colorectal pathologies and prognostic factors for CRC [45, 46]. Interestingly, down-regulated genes associated with “Cell adhesion molecules (CAMs)”, known to play a fundamental role in the metastatization of CRC when down-regulated; “regulation of BMP signaling pathway”, whose inactivation is consolidated in the initiation/progression of gastrointestinal cancers; “Formation of NR-MED1 Coactivator Complex”, often down-regulated in CRC, linked to the WNT/β-catenin signaling cascade, and associated with lymph node metastasis and advanced TNM stage [47–53].

Due to the general characterization supplied in this work, we did not evaluate the functionality of non-coding genes (27% of DEGs) on which we will focus in future works. However, as a proof of principle, we provided a detailed characterization of differentially expressed non-coding transcript biotypes identified in the LRT Late versus Early comparison (Supplementary Table 3). Specifically, focusing on the two up-regulated miRNAs, we found no validated target genes (TarBase v8.0, last accessed: April 12, 2021) for hsa-miR-6829 (log2FC = 2.13, padj = 0.009), a poorly characterized miRNA whose role, so far, has only been implied in asthma (rs2448519, [54]); on the contrary, hsa-miR-221 (log2FC = 2.3, padj = 0.026) regulates 926 and 848 validated targets with its mature forms hsa-miR-221-3p and hsa-miR-221-5p, respectively. MiR-221 is a known onco-miRNA in CRC cells [55] and we recently confirmed that it can regulate the tumor suppressor PTEN expression [56]. For this reason, we examined how many validated targets of hsa-miR-221-3p (*n* = 926) and hsa-miR-221-5p (*n* = 848) were also interacting with PTEN (*n* = 174; Harmonizome Pathway Commons Protein-Protein Interactions [57], last accessed: April 12, 2021), finding fifteen and seventeen targets, respectively, with two genes (CTNNB1 and DBN1) regulated by both mature miRNAs. We finally checked their expression status in the LRT Late versus Early comparison, and found that they were both down-regulated, as expected, although in a mild way (log2Fc − 0.43 and − 0.71; padj < 0.05), providing additional layers of regulation on the PTEN axis.

### Phosphoprotein-based signaling profiles of patient-derived organoids

**Fig. 4:**
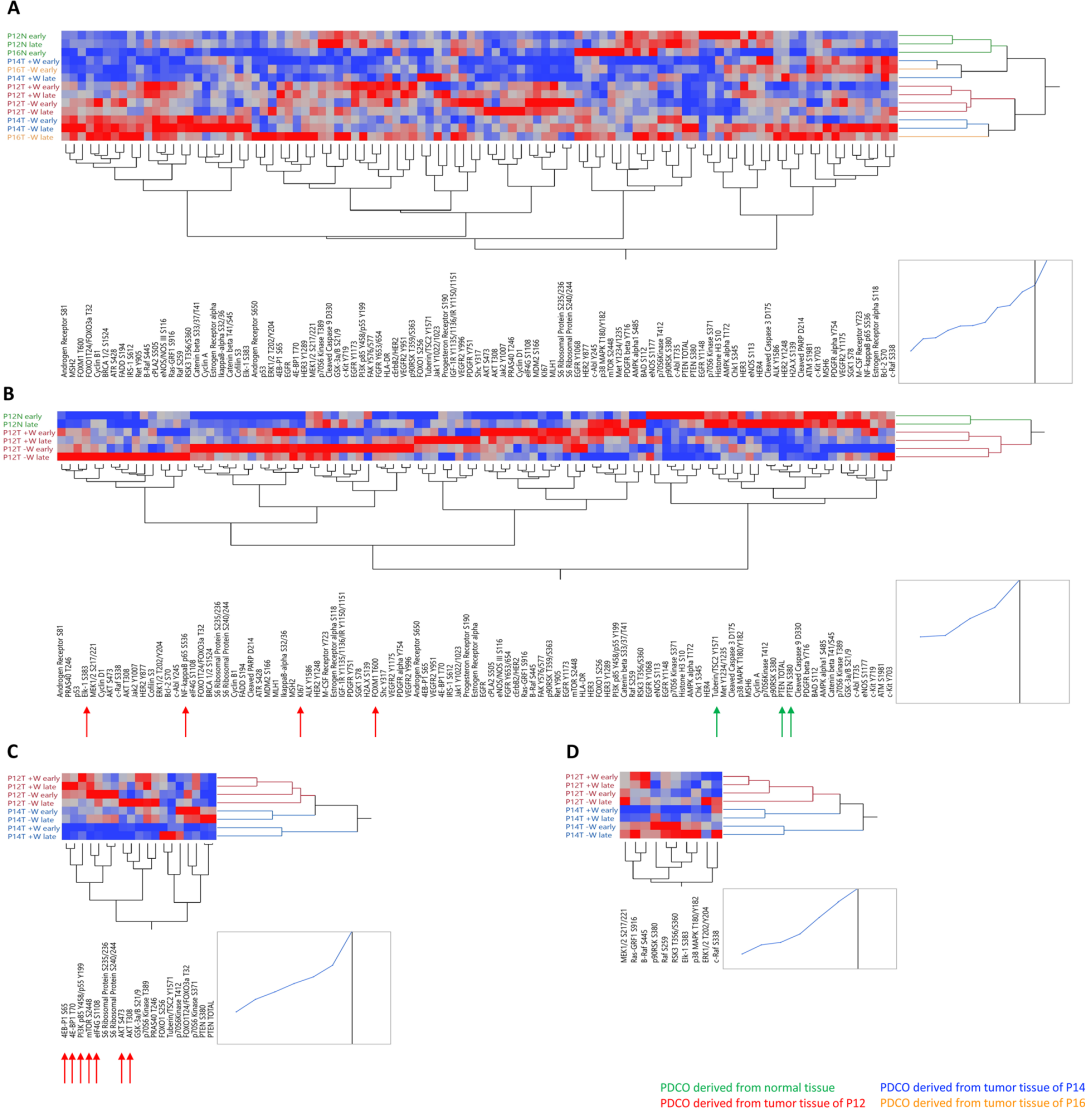
RPPA-based signaling network analyses of tumor/normal Early/Late PDCOs. **A**, **B** Unsupervised hierarchical clustering analyses illustrating changes in expression and/or activation of the analytes measured by RPPA across all samples (A) and in P12 PDCOs derived from normal and matched tumor cells (B). **C**, **D** Target analyses of the PI3K/AKT/mTOR and the MAPK signaling pathway of Early/Late tumor-derived PDCOs for patients P12 and P14 are shown, respectively. Proteins with missing values were not included in the unsupervised analyses

To define changes in signaling events within and across PDCOs established from patients P12, P14 and P16, we used the Reverse Phase Protein Microarray (RPPA) to capture expression and/or post-translational modifications of 120 proteins belonging to different signaling pathways known to be involved in cancer onset and progression. Unsupervised hierarchical clustering revealed heterogeneous profiles across models. PDCOs derived from normal-appearing tissue from patients P12 and P16 were contained within the same cluster. Early and later passages of the P12-derived samples showed a greater level of similarity compared to P16 (Fig. 4A). Interestingly, in the P12 PDCOs expression and/or post-translationally modified tumor suppressors PTEN and TSC2 were increased in the normal-appearing samples compared to the malignant one. These changes in PTEN expression level and stability may be attributable to the acquisition of a pathogenic PTEN mutation (R335X) in the tumor-derived models. On the contrary, expression of the proliferation marker Ki67 along with post-translationally modified transcription factors like FOXM1 (T600), Elk-1 (S383), and NF-kappaB p65 (S536) were increased in PDCOs derived from malignant lesions compared to the normal counterpart (Fig. 4B).

Compared to the PDCOs derived from other tumors, P12-derived samples were contained within the same cluster suggesting that profiles are overall maintained across samples derived from the same patient regardless of media composition and collection time (early versus late passages). Similarly, the number of passages did not appear to affect the molecular profiles of the P14-derived models indicating that molecular events are relatively stable over time in PDCOs (Fig. 4A).

We next explored the impact of PIK3CA and PTEN mutations on the activation of the PI3K/AKT/mTOR signaling pathway in P12 and P14 (Fig. 4C and Supplementary Fig. 7). Interestingly, when different phosphorylation sites of the same proteins were measured, high levels of concordance were detected for multiple members of the PI3K/AKT/mTOR pathway (e.g. AKT, 4EBP1, S6 Ribosomal Proteins), confirming the activation of these key substrates. As expected, PDCOs for patient P12, which harbored pathogenic mutations of PIK3CA and PTEN, presented greater phosphorylation levels of PI3K p85 (Y458)/p55 (Y199) and the downstream substrates AKT (S473 & T308), mTOR (S2448), and 4EBP1 (S65 & T70) compared to P14 (Fig. 4C). Finally, we compared *KRAS* allele-specific activation of the MAPK signaling pathway in PDCOs derived from P12 and P14, harboring a G12V and G13C KRAS mutation, respectively. As shown in Fig. 4D, MAPK downstream signaling events, including phosphorylation of ERK substrates Elk-1 (S383), p38 MAPK (T180/Y182), p90RSK (S380), RSK3 (T356/S360) were greater in sample P14 compared to P12, when unmodified media was used.

Signal transduction analyses, across the three models, identified heterogeneous responses in PDCOs based on the condition media used and time of sample collection. While the impact was minimal for P12T-derived models, P14T cells clustered together based on the growth media used. These different behaviors may be driven by distinct susceptibility and baseline signal transduction activity in each model. For example, the activation levels of members of the WNT signaling pathway may affect the response to each specific growth condition. However, the effect of the composition of the media for the P14T model was maintained across time as early and late samples were grown under the same conditions clustered together, suggesting that personalized molecular profiles could modulate the response to the different growing conditions.

## Discussion

CRC is the fourth cause of cancer-related death in the world, leading to approximately 10% of worldwide mortality [1–3]. CRC is mostly surgically resected, however, the (neo)adjuvant therapy is often used to avoid a relapse. Although chemoradiotherapy has decreased the risk of CRC recurrence, metastatic CRC requires the combined use of chemotherapeutic agents and targeted therapy and immunotherapy [58]. Nevertheless, the multiple-treatments regimen depends on the specific tumor molecular profile. Patient-derived tumor organoids are an emerging 3D-cell model to study the tumor identity for personalized medicine. Indeed, the possibility to mimic the tumor microenvironment allows to develop reliable cancer models, representing a powerful tool for the transferability of pre-clinical models to the patient [59]. Recently, the characterization of single-cell post-translational modifications has revealed the oncogenic mutations involved in signaling networks in CRC murine organoids co-cultured with stromal and immune cells [52], emphasizing the necessity of complementary experimental approaches to fully recapitulate all the intertwined regulatory events occurring during tumorigenesis and tumor expansion. In literature, a detailed pipeline combining next-generation sequencing and proteomic analyses on patient-derived organoids in different culturing conditions is missing. Noteworthy, this aspect is necessary in order to transfer the use of patient-derived 3D-cell model from benchtop to bedside, and *vice versa*, to implement personalized therapies. Here, we methodically characterized PDCOs at the molecular level, under different culturing conditions, by combining genomic, transcriptomic, and proteomic profiles, to define PDCOs aptitude to stably recapitulate patient-derived tissues.

Although specific signatures can be defined at different molecular levels, uniquely characterizing normal and tumor organoids, it is well-consolidated that variability is intrinsic in almost every examined specimen [18, 25], leading to different outcomes in terms of, among all, patient survival and drug response. Our observations suggest that the baseline molecular profile of individual models may modulate their susceptibility and adaptation to different growing conditions. Understanding which condition better recapitulates the molecular events of the primary tumors may guide the choice of the most appropriate model for each patient. Due to the limited amount of clinical material available for this analysis and the need for upfront cellular enrichment to generate molecular profiles uniquely attributable to tumor cells, a direct comparison between PDCOs and matched primary tumors will be the focus of future investigations.

The expansion of a more aggressive subset of cancer cells, however, is still promoted and allows to accurately study the temporal evolution of tumors. This is corroborated by the widespread increase in the VAF of observed genomic variants and by the identification of a novel PTEN mutation. To the best of our knowledge, this variant has never been detected before, not even in the whole TCGA datasets (*n* = 32, “Firehose Legacy”, last accessed: April 12, 2021), indicating that this mutation most likely represents a rare event in cancer.

The transcriptional profiling of PDCOs confirmed the differential expression of CRC markers (e.g. MUC1, MUC4 and CA2 up-regulated in normal organoids; PROX1, BAMBI, PTCH1 and APCDD1 up-regulated in tumor organoids) [18] but applying the likelihood ratio test also allowed to study combined changes induced by time and condition, finding for instance that the APCDD1 up-regulation, compared to normal organoids, is progressively reduced during tumor PDCOs development.

By combining a multi-omics approach, in which results confirm or complement each other [25, 60], altered pathways in CRC progression were efficiently highlighted. Thus, we propose a powerful pipeline that can be exploited as a pre-clinical model, guiding to optimized individual treatments based on molecular profiling and avoiding delays in the use of the most efficient therapeutic regimens.

## Conclusions

To define a successful cancer therapy befitting individual heterogeneity, employing combined next-generation sequencing and proteomic analysis on CRC organoids is confirmed to be a very promising tool for personalized medicine, representing a powerful instrument from benchtop to bedside. Although the number of analyzed patients is limited, the amount of experimental growth and timing conditions, as well as the large amount of data obtained by the integrated multi-omics pipeline used for molecular analysis, allowed a meticulous characterization of the 3D model.

Nevertheless, increasing the number of specimens collected in our databank is our top priority. This will increase the robustness of our data and, at the same time, will allow the characterization of patients in subtypes based on existing (e.g. TCGA) gene signatures [61]. Additionally, more samples will be also required to test drug sensitivity of organoids based on the assumption that molecules acting on the same targets would act similarly on the PDCOs collection, clustering based on their IC50 [18].

Overall, our data strongly support the use of CRC organoids as a novel, very promising tool for personalized medicine that could represent a powerful alternative to animal experimentation in vivo, opening new perspectives for the identification of novel biomarkers of cancers.

## Supplementary Information

**Additional file 1.**

## Data Availability

The datasets supporting the conclusions of this article are available in the NCBI’s Gene Expression Omnibus (Edgar et al., 2002) repository, GSE157004 (https://www.ncbi.nlm.nih.gov/geo/query/acc.cgi?acc=GSE157004). For reviewers, please enter the following token: spqveyaknzorrwv.

## Abbreviations

3D: Three-dimensional
abs: Absolute
ACMG: American College of Medical Genetics and Genomics
AF: Allele frequency
AIRC: Associazione Italiana per la Ricerca sul Cancro
AMP: Association for Molecular Pathology
BER: Base excision repair
BWA: Burrows-Wheeler aligner
CAMs: Cell adhesion molecules
CIMP: CpG island methylator phenotype
CIN: Chromosomal instability
COSMIC: Catalogue Of Somatic Mutations In Cancer
CRC: Colorectal cancer
DDR: DNA damage response
DEGs: Differentially expressed genes
FC: Fold change
FDR: False discovery rate
gDNA: Genomic DNA
GEO: Gene expression omnibus
HGNC: HUGO gene nomenclature committee
Indels: Insertions and deletions
LOH: Loss of heterozygosity
LRT: Likelihood ratio test
MAF: Minor allele frequency
MMR: Mismatch repair
MSI: Microsatellite instability
PDCOs: Patient-derived colon organoids
RBC: Red Blood Cell
RPPA: Reverse-phase protein microarrays
RT: Room temperature
SNVs: Single nucleotide variants
VAF: Variant allele fraction
WES: Whole-exome sequencing
