# Supplementary material for: Integrated multi-omics analyses on patient-derived CRC organoids highlight altered molecular pathways in colorectal cancer progression involving PTEN
Source: J Exp Clin Cancer Res. 2021 Jun 21;40:198. doi: 10.1186/s13046-021-01986-8 (PMC8215814; doi:10.1186/s13046-021-01986-8)

Supplementary Figure 6

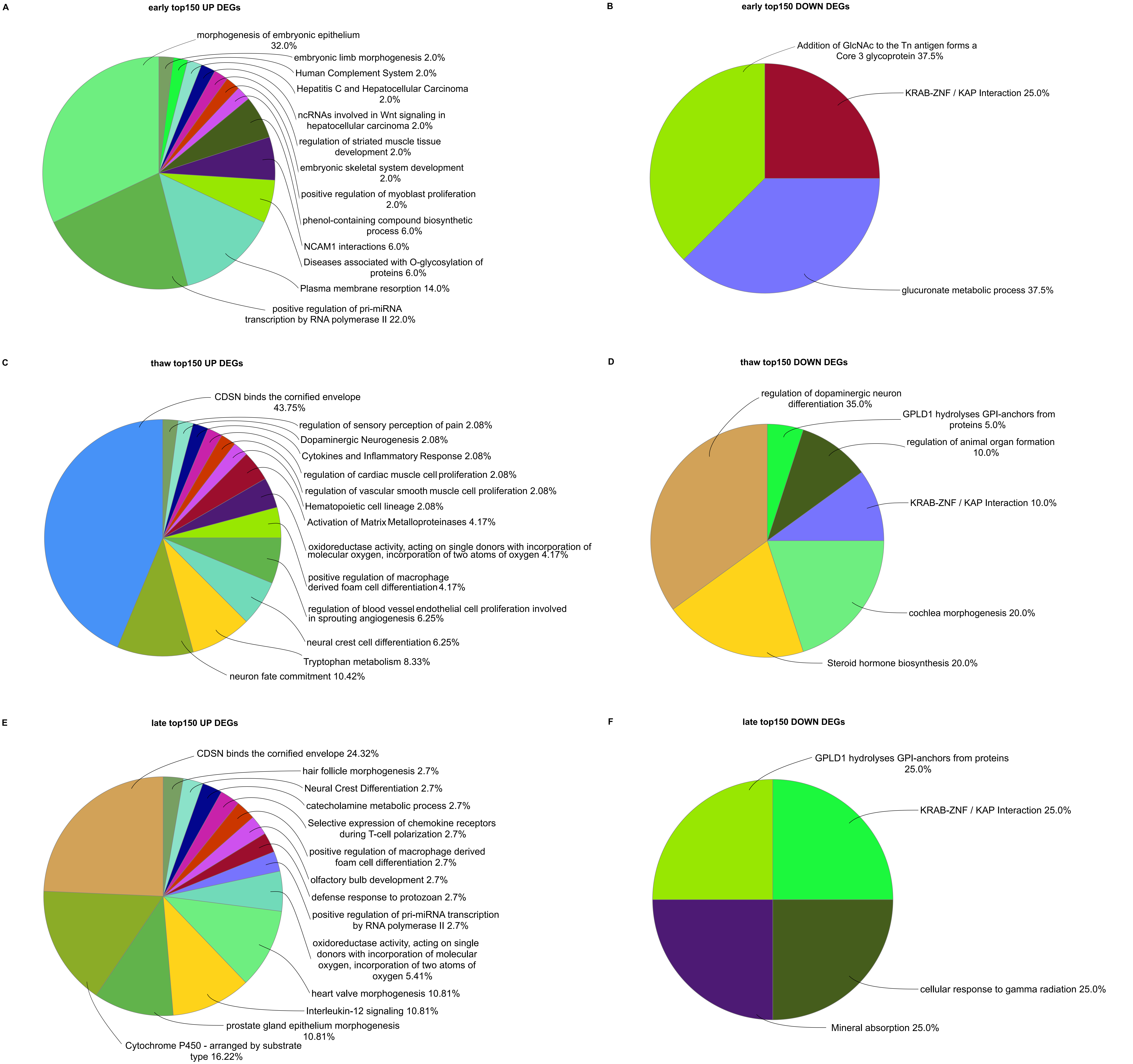

Supplement: Supplementary file 1 — Additional file 1. [file 13046_2021_1986_MOESM1_ESM.zip › Supplementary Figure 6.pdf]
