# Supplementary material for: Integrated multi-omics analyses on patient-derived CRC organoids highlight altered molecular pathways in colorectal cancer progression involving PTEN
Source: J Exp Clin Cancer Res. 2021 Jun 21;40:198. doi: 10.1186/s13046-021-01986-8 (PMC8215814; doi:10.1186/s13046-021-01986-8)
Supplement: Supplementary file 1 — Additional file 1. [file 13046_2021_1986_MOESM1_ESM.zip › Codrich et al Supplementary Data_revised_Final.docx]

**Supplementary Information**

**Integrated multi-omics analyses on patient-derived CRC organoids highlight altered molecular pathways in colorectal cancer progression involving PTEN**

Marta Codrich, Emiliano Dalla, Catia Mio, Giulia Antoniali, Matilde Clarissa Malfatti, Stefania Marzinotto, Mariaelena Pierobon, Elisa Baldelli, Carla Di Loreto, Giuseppe Damante, Giovanni Terrosu, Carlo Pucillo, and Gianluca Tell*

**Supplementary Materials and Methods**

**Organoid normal culture.** The generation of patient-derived normal organoids was performed as described by ^1^ with some modifications. Normal colon tissue was washed with ice-cold PBS supplemented with penicillin (100 U*mL^-1^) and streptomycin (10 μg*mL^-1^) (EuroClone) several times. The epithelial layer was dissected from muscle and fat layers and cut into around 2 mm pieces. The tissue fragments were washed with PBS supplemented with penicillin (100 U*mL^-1^), streptomycin (10 μg*mL^-1^) (EuroClone) and DTT (0.5 mM). Tissue fragments were subsequently incubated in PBS supplemented with penicillin (100 U*mL^-1^), streptomycin (10 μg*mL^-1^) (EuroClone), DTT (0.5 mM) and EDTA (10 mM) (Sigma-Aldrich). The suspension was rotated for 1 hour at 4°C. After incubation, crypts were isolated from the supernatant by pipetting. Supernatant was transferred into a new tube containing FBS (100%) (BioWest). The solution containing the crypts was centrifuged at 800 rpm for 5 min at 4°C. Crypts were resuspended in basal medium, composed of Advanced DMEM/F12 (Life Technologies, Carlsbad, CA, USA) supplemented with Glutamax (2 mM) (Life Technologies), HEPES (10 mM) (Life Technologies), penicillin (100 U*mL^-1^) and streptomycin (10 μg*mL^-1^) (EuroClone). Crypts were counted and 500 crypts/10μL were mixed with Matrigel (20 μL) (Corning) and 3 drops (10 μL each) were plated in a single well of a 24-well plate. After polymerization of Matrigel (Corning®, Corning, NY, USA) for 10-15 min at 37°C, culture medium was added (500 µl). Normal organoids were cultured in basal medium containing 1X B27 (Life Technologies), N-acetylcysteine (1.15 mM) (Sigma-Aldrich), Nicotinamide (10 mM) (Sigma-Aldrich), Gastrin I (10 nM) (Tocris Bioscience, Bristol, UK), Prostaglandin E2 (10 nM) (Sigma-Aldrich), A83-01 (500 nM) (R&D System, Minneapolis, MN, USA), mEGF (50 ng*mL^-1^) (PeproTech, London, UK), SB202190 (3 µM) (Sigma-Aldrich), Wnt3a Conditioned Medium (50%), Noggin Conditioned Medium (10%), R-Spondin1 Conditioned Medium (20%), Y-27632 (10 µM (Abcam) and Primocin (100 μg*mL^-1^) (InvivoGen). The medium was refreshed every two/three days. Normal organoids were passaged 1:2 every one/two weeks.

**Organoid tumor culture.** The generation of patient-derived tumor organoids was performed as described by ^2^ with some modifications. Tumor colon tissue was washed with ice-cold PBS supplemented with penicillin (100 U*mL^-1^) and streptomycin (10 μg*mL^-1^) (EuroClone) several times. Tumor tissue was homogenized with scissors and then digested with Liberase (0.26 U*mL^-1^) (Sigma-Aldrich, St. Louis, MO, USA) in basal medium, composed of Advanced DMEM/F12 (Life Technologies, Carlsbad, CA, USA) supplemented with Glutamax (2 mM) (Life Technologies), HEPES (10 mM) (Life Technologies), penicillin (100 U*mL^-1^) and streptomycin (10 μg*mL^-1^) (EuroClone) complemented with Primocin (100 μg*mL^-1^) (InvivoGen, San Diego, CA, USA) and Y-27632 (10 µM) (Abcam, Cambridge, UK) for 1 hour at 37 °C shaking at 250 rpm. The resulting fraction was passed through a 100-mm cell strainer. The filtered solution was centrifuged at 1,200 rpm for 5 min at 4°C. The cell pellet was resuspended with Red Blood Cell (RBC) Lysis Buffer (BioLegend, San Diego, CA, USA) and incubated for 10 min at room temperature (RT) in the dark. RBC buffer was neutralized adding basal medium complemented with fetal bovine serum (10%) (BioWest, Nuaillé, France). The resulting solution was centrifuged at 1,200 rpm for 5 min at 4°C and the cell pellet was resuspended in basal medium. Cells were counted with a Burker chamber and 200,000 cells/10 μL were mixed with Matrigel (20 μL) and 30 μL drop was plated in a single well of a 24-well plate. After polymerization of Matrigel (Corning®, Corning, NY, USA) for 10-15 min at 37°C, culture medium was added (500 µl). Tumor organoids were cultured in basal medium containing B27 (1X) (Life Technologies), N-acetylcysteine (1.15 mM) (Sigma-Aldrich), Nicotinamide (10 mM) (Sigma-Aldrich), Gastrin I (10 nM) (Tocris Bioscience, Bristol, UK), Prostaglandin E_2_ (10 nM) (Sigma-Aldrich), A83-01 (500 nM) (R&D System, Minneapolis, MN, USA), mEGF (50 ng mL^-1^) (PeproTech, London, UK), SB202190 (3 µM) (Sigma-Aldrich), Noggin Conditioned Medium (10%), R-Spondin1 Conditioned Medium (20%), Y-27632 (10 µM) (Abcam) and Primocin (100 μg mL^-1^) (InvivoGen) with or without Wnt3a Conditioned Medium (50%). The medium was refreshed every two/three days. Tumor organoids were passaged 1:4 every one/two weeks. The morphology of PDCOs did not undergo any significant modifications among the different conditions.

**Somatic mutations calling.** The Burrows-Wheeler Aligner (BWA) ^3^ was used to align reads to the NCBI GRCh38 human genome reference assembly. Samtools ^4^ and Picard (<http://broadinstitute.github.io/picard/>) were used for data conversion, indexing and manipulation. Somatic single nucleotide variants (SNVs), insertions and deletions (indels) were called via local assembly of haplotypes following the GATK somatic short mutation calling best practice workflow (<https://gatk.broadinstitute.org/hc/en-us/articles/360035531132>), using the Mutect2 method ^5^ on paired tumor-normal samples and applying the recommended settings. Variants that passed the filtering steps were annotated using the ANNOVAR software tool ^6^, retaining those deemed deleterious according to SIFT or CLINVAR and those with an undefined role that were non-synonymous and mapped to gene exons, splicing junctions or UTRs.

**RNA sequencing and data analysis.** Sequencing of TruSeq Stranded mRNA samples was carried out on paired-end 101 bp mode on NextSeq 500 (Illumina, San Diego, CA). Sequencing reads quality was evaluated using the ShortRead (v1.44.3) R/Bioconductor package ^7^. Quality, adapters and contamination filtering were performed using the Trimmomatic ^8^ command-line tool. Processed reads were aligned to the NCBI GRCh38 human reference using STAR (v2.7.1a) ^9^. Transcript assembly and the number of reads per gene were determined using Stringtie (v1.3.6) ^10^. Differentially expressed genes were identified using the DESeq2 (v1.26.0) R/Bioconductor package ^11^, considering as statistically significant the results having abs(log2FC)≥1, FDR < 0.05. Extended gene annotations (including HGNC gene symbol, description and transcript type) were obtained using the biomaRt (v2.42.0) R/Bioconductor package ^12^. The sample-to-sample distance matrix was generated applying the dist function of the stats (v3.6.3) R/Bioconductor package to the transpose of the vsd transformed count matrix. The pheatmap (v1.0.12) R/Bioconductor package was used for heatmap visualization ^13^. Heatmaps and hierarchical clustering of gene expression data were obtained using the cluster_rows and cluster_cols arguments of the pheatmap package, using the Euclidean distance and the complete agglomeration method. The principal components analysis was computed using R/Bioconductor and the results were plotted using the rgl package on the vst transformed count matrix ^14^. The functional enrichment analysis was performed using the Cytoscape plugin ClueGO to identify enriched terms ^15,16^. The following functional databases were queried: CLINVAR_Human-diseases (08.05.2020), WikiPathways (08.05.2020), KEGG (08.05.2020), REACTOME_Reactions (08.05.2020), REACTOME_Pathways (08.05.2020), GO_ImmuneSystemProcess (08.05.2020), GO_BiologicalProcess (08.05.2020) and CORUM_CORUM-FunCat-MIPS (03.09.2018). Default parameters were applied. A two-sided hypergeometric test (corrected using the Benjamini-Hochberg method to control the false discovery rate, adjusted p ≤ 0.05) was used to determine the probability that each functional term was assigned to the gene sets due to chance alone. Data handling was performed in R (versions 3.6.1 and 3.6.2) using RStudio (v1.2).

**Supplementary Tables**

**Supplementary Table 1**. Overview of patients’ profile. Profile of the patients (P12, P14 and P16) affected by CRC analyzed in this study.

| **Patient** | **Age** | **Sex** | **Neoplasia site** | **Histology** | **Grading** | **Histological stage** | **Metastasis** | **ICD-O** | |
| --- | --- | --- | --- | --- | --- | --- | --- | --- | --- |
| P12 | 77 | F | Right Colon | Mucinous carcinoma | 3 | pT3N2bM1a | Liver | 1538 | 4573 |
| P14 | 79 | M | Right Colon | Adenocarcinoma | 2 | pT3N1aM1 | - - - | 1536 | 4573 |
| P16 | 79 | M | Right Colon | Adenocarcinoma | 2 | pT3N1bM0 | - - - | 1536 | 4573 |

**Supplementary Table 2**. Overview of the collection of the primary tissue and PDCOs samples. For each patient, we indicated the type of tissue or patient-derived colon organoids (PDCOs) (normal N or tumor T), the growing condition, the timing of collection (stage, passage and days) and the type of analysis performed (whole-exome sequencing (WES), microsatellite profile, Targeted NGS, RNAseq, histochemistry and RPPA).

| **P** | **N/T** | **S** | **M** | **WES** | | | **Microsatellite profile** | | | **Targeted NGS** | | | **RNAseq** | | | **Histochemistry** | | | **RPPA** | | |
| --- | --- | --- | --- | --- | --- | --- | --- | --- | --- | --- | --- | --- | --- | --- | --- | --- | --- | --- | --- | --- | --- |
|  |  |  |  | **St** | **Pass** | **D** | **St** | **Pass** | **D** | **St** | **Pass** | **D** | **St** | **Pass** | **D** | **St** | **Pass** | **D** | **St** | **Pass** | **D** |
| **P12** | N | primary tissue | / | 0 | 0 | 0 | 0 | 0 | 0 | 0 | 0 | 0 | - - - | - - - | - - - | - - - | - - - | - - - | - - - | - - - | - - - |
|  | T | primary tissue | / | 0 | 0 | 0 | 0 | 0 | 0 | 0 | 0 | 0 | - - - | - - - | - - - | 0 | 0 | 0 | - - - | - - - | - - - |
|  | N | PDCO | +W | e | p4 | 40 | e | p4 | 40 | e | p4 | 40 | e | p3 | 21 | e | p7 | 56 | e | p7 | 56 |
|  | N | PDCO | +W | - - - | - - - | - - - | - - - | - - - | - - - | - - - | - - - | - - - | t | p9 | 56 | - - - | - - - | - - - | - - - | - - - | - - - |
|  | N | PDCO | +W | l | p13 | 91 | l | p13 | 91 | - - - | - - - | - - - | l | p13 | 95 | l | p11 | 85 | l | p11 | 85 |
|  | T | PDCO | +W | e | p4 | 55 | e | p4 | 55 | - - - | - - - | - - - | - - - | - - - | - - - | e | p5 | 63 | e | p5 | 63 |
|  | T | PDCO | +W | - - - | - - - | - - - | - - - | - - - | - - - | - - - | - - - | - - - | - - - | - - - | - - - | l | p12 | 88 | l | p12 | 88 |
|  | T | PDCO | -W | e | p4 | 55 | e | p4 | 55 | e | p4 | 55 | e | p4 | 21 | e | p6 | 62 | e | p6 | 62 |
|  | T | PDCO | -W | - - - | - - - | - - - | - - - | - - - | - - - | - - - | - - - | - - - | t | p9 | 56 | - - - | - - - | - - - | - - - | - - - | - - - |
|  | T | PDCO | -W | l | p12 | 87 | l | p12 | 87 | - - - | - - - | - - - | l | p12 | 95 | l | p12 | 81 | l | p12 | 81 |
| **P14** | N | primary tissue | / | 0 | 0 | 0 | 0 | 0 | 0 | 0 | 0 | 0 | - - - | - - - | - - - | - - - | - - - | - - - | - - - | - - - | - - - |
|  | T | primary tissue | / | 0 | 0 | 0 | 0 | 0 | 0 | 0 | 0 | 0 | - - - | - - - | - - - | 0 | 0 | 0 | - - - | - - - | - - - |
|  | N | PDCO | +W | e | p5 | 50 | e | p5 | 50 |  | p5 | 50 | - - - | - - - | - - - | - - - | - - - | - - - | - - - | - - - | - - - |
|  | T | PDCO | +W | e | p5 | 61 | e | p5 | 61 | - - - | - - - | - - - | - - - | - - - | - - - | e | p7 | 65 | e | p7 | 65 |
|  | T | PDCO | +W | l | p12 | 113 | l | p12 | 113 | - - - | - - - | - - - | - - - | - - - | - - - | - - - | - - - | - - - | l | p12 | 131 |
|  | T | PDCO | +W | vl | p17 | 150 | vl | p17 | 150 | - - - | - - - | - - - | - - - | - - - | - - - | - - - | - - - | - - - | - - - | - - - | - - - |
|  | T | PDCO | -W | e | p5 | 61 | e | p5 | 61 | e | p5 | 61 | - - - | - - - | - - - | e | p6 | 67 | e | p6 | 67 |
|  | T | PDCO | -W | l | p12 | 94 | l | p12 | 94 | - - - | - - - | - - - | - - - | - - - | - - - | l | p12 | 125 | l | p12 | 125 |
| **P16** | N | primary tissue | - - - | - - - | - - - | - - - | - - - | - - - | - - - | - - - | - - - | - - - | - - - | - - - | - - - | - - - | - - - | - - - | - - - | - - - | - - - |
|  | T | primary tissue | / | - - - | - - - | - - - | - - - | - - - | - - - | - - - | - - - | - - - | - - - | - - - | - - - | 0 | 0 | 0 | - - - | - - - | - - - |
|  | N | PDCO | - - - | - - - | - - - | - - - | - - - | - - - | - - - | - - - | - - - | - - - | - - - | - - - | - - - | e | p7 | 61 | e | p7 | 61 |
|  | T | PDCO | -W | - - - | - - - | - - - | - - - | - - - | - - - | - - - | - - - | - - - | - - - | - - - | - - - | - - - | - - - | - - - | e | p5 | 33 |
|  | T | PDCO | -W | - - - | - - - | - - - | - - - | - - - | - - - | - - - | - - - | - - - | - - - | - - - | - - - | l | p12 | 118 | l | p12 | 118 |

P: Patient; S: Sample; M: Medium; St: Stage; Pass: Passage; D: Days; e: early; l: late; vl: very late; t: thaw

**Supplementary Table 3.** Summary of the differentially expressed, non-coding RNA biotypes profiled with the likelihood ratio test (LRT) in the Late Vs Early comparison of tumor PDCOs derived from patient P12 (-W medium) versus normal PDCOs.

| **Class** | **Transcript biotype** | **Occurrences** |
| --- | --- | --- |
| **lncRNA** |  | **437** |
|  | lncRNA | 405 |
|  | lncRNA,retained_intron | 28 |
|  | retained_intron,lncRNA | 4 |
| **Pseudogene** |  | **167** |
|  | IG_V_pseudogene | 2 |
|  | polymorphic_pseudogene | 1 |
|  | processed_pseudogene | 91 |
|  | processed_transcript,retained_intron,transcribed_unprocessed_pseudogene | 2 |
|  | processed_transcript,transcribed_processed_pseudogene | 13 |
|  | processed_transcript,transcribed_unitary_pseudogene | 4 |
|  | processed_transcript,transcribed_unitary_pseudogene,retained_intron | 1 |
|  | processed_transcript,transcribed_unprocessed_pseudogene | 30 |
|  | processed_transcript,transcribed_unprocessed_pseudogene,retained_intron | 1 |
|  | retained_intron,processed_transcript,transcribed_unitary_pseudogene | 1 |
|  | rRNA_pseudogene | 1 |
|  | transcribed_processed_pseudogene | 1 |
|  | transcribed_processed_pseudogene,processed_transcript | 2 |
|  | transcribed_unprocessed_pseudogene | 2 |
|  | transcribed_unprocessed_pseudogene,retained_intron,processed_transcript | 1 |
|  | translated_processed_pseudogene | 1 |
|  | unprocessed_pseudogene | 13 |
| **miRNA** |  | **2** |
| **snoRNA** |  | **2** |
| **misc RNA** |  | **60** |
|  | misc_RNA | 4 |
|  | nonsense_mediated_decay | 5 |
|  | nonsense_mediated_decay,retained_intron | 1 |
|  | processed_transcript,nonsense_mediated_decay,retained_intron | 1 |
|  | NA | 46 |
|  | TEC | 1 |
|  | retained_intron | 2 |

**Supplementary Figures**

**Supplementary Figure 1.** Brightfield representative images of PDCOs derived from different patients (P12 and P14) cultured with (+W) or without (-W) the Wnt3a factor at different passages. Magnification 5X.

**Supplementary Figure 2.** Hematoxylin eosin staining and immunohistochemical staining on CRC samples and the corresponding PDCOs**. A,** Hematoxylin eosin staining on CRC samples and the corresponding PDCOs cultured with (+W) or without the Wnt3a factor (-W) at different stages. Scale bar 100 μm. **B,** Immunohistochemical staining for the MLH1, MSH2, MSH6 and PMS2 proteins of patients P12, P14 and P16. Paraffin-embedded tissue and PDCOs sections of CRC samples were stained with antibodies for the MLH1, MSH2, MSH6 and PMS2 proteins. Scale bar 100 μm.

**Supplementary Figure 3.** Microsatellite profile of PDCOs. Fragment analysis of normal and tumor DNA of primary tissue and PDCOs for patient 12 (A) and 14 (B). Each peak is visualized with the corresponding marker. **A,** All the markers showed microsatellite stability in patient 12. **B,** D5S346, CSF1PO and D18S51 showed microsatellite instability in patient 14, whereas BAT26, D17S250, TGFBR2, D2S123, BAT25, D18S58 MT1XT20, D7S820 and BAT40 were stable.

**Supplementary Figure 4.** Sanger sequencing evaluating PTEN c.806_817dup. Sanger sequencing confirmation of NGS data showing the 12 bp duplication (black line) in *PTEN* exon 8 harbored by the P14T -W early.

**Supplementary Figure 5. A,** Principal Components Analysis of the RNA-seq data. The vst-transformed RNA-seq counts matrix was used to perform the analysis. The first three principal components are shown and each experimental condition is represented using a distinct color. The profiled samples are grouped in two main clusters, corresponding to tumor PDCOs derived from patient P12 (-W medium; on the right) and to normal PDCOs (on the left). **B-D,** Differential gene expression in tumor *versus* normal PDCOs at early, thaw and late time points. Heatmaps showing differentially expressed transcripts applying the Wald test to evaluate normal and tumor PDCOs gene expression levels. Globally, 5,657 DEGs were found at early time point (B) (3,224 up-regulated and 2,433 down-regulated genes), 4,613 DEGs at that time point (C) (2,833 up- and 1,780 down-regulated) and 5,290 DEGs at late time point (D) (2,746 up- and 2,544 down-regulated) (abs(log2FC)≥1, FDR < 0.05). Hierarchical clustering of transcripts was performed using the Euclidean distance and the complete agglomeration method; gene expression data was vst-transformed, scaled and centered. **E-G,** Examples of LRT differentially expressed genes. The likelihood ratio test (LRT) allows identifying condition-specific differences over time. Plot of gene expression changes over time of the most up-regulated tumor PDCOs gene (E) (KLHL30, log2FC=12.2, padj=5.29e-08). Plot of gene expression changes over time of the most down-regulated tumor PDCOs gene (F) (SLC8A3, log2FC=-11.8, padj=2.04e-06). Plot of gene expression changes over time of a not significant differentially expressed tumor PDCOs gene (G) (TTLL4, log2FC= 0.00017, padj=1).

**Supplementary Figure 6.** Functional enrichment analysis of differentially expressed genes at the early, thaw and late time points. The top150 significantly up- (**A, C, E**) and down-regulated (**B, D, F**) coding genes identified applying the Wald test to normal and tumor PDCOs gene expression data at the early, thaw and late time points were annotated using the Cytoscape plugin ClueGO. Functionally enriched terms (Benjamini-Hochberg adjusted p ≤ 0.05) were identified querying the CLINVAR_Human-diseases, WikiPathways, KEGG, REACTOME_Reactions, REACTOME_Pathways, GO_ImmuneSystemProcess, GO_BiologicalProcess and CORUM_CORUM-FunCat-MIPS databases. Pie chart colors correspond to the different enriched functional clusters, the most significant term of each cluster used as cluster representative and identifier.

**Supplementary Figure 7.** Correlation between PTEN expression and AKT S473 activation across PDCO models. Bar graph showing the RPPA intensity values for PTEN expression and matched AKT S473 activation in PDCOs derived from normal and tumor tissue from P12, P14, and P16. PTEN mutation in tumor-derived PDCO models from P12 were associated with reduce PTEN expression and consequent increased phosphorylation levels of AKT S473 compared to the normal counterpart and to the PTEN wild-type P14 and P16.

**Supplementary References**

1 Van De Wetering M, Francies HE, Francis JM, Bounova G, Iorio F, Pronk A *et al.* Prospective derivation of a living organoid biobank of colorectal cancer patients. *Cell* 2015; **161**: 933–945.

2 Fujii M, Shimokawa M, Date S, Takano A, Matano M, Nanki K *et al.* A Colorectal Tumor Organoid Library Demonstrates Progressive Loss of Niche Factor Requirements during Tumorigenesis. *Cell Stem Cell* 2016; **18**: 827–838.

3 Li H, Durbin R. Fast and accurate short read alignment with Burrows-Wheeler transform. *Bioinformatics* 2009; **25**: 1754–1760.

4 Li H, Handsaker B, Wysoker A, Fennell T, Ruan J, Homer N *et al.* The Sequence Alignment/Map format and SAMtools. *Bioinformatics* 2009; **25**: 2078–2079.

5 Cibulskis K, Lawrence MS, Carter SL, Sivachenko A, Jaffe D, Sougnez C *et al.* Sensitive detection of somatic point mutations in impure and heterogeneous cancer samples. *Nat Biotechnol* 2013; **31**: 213–219.

6 Wang K, Li M, Hakonarson H. ANNOVAR: Functional annotation of genetic variants from high-throughput sequencing data. *Nucleic Acids Res* 2010; **38**. doi:10.1093/nar/gkq603.

7 Morgan M, Anders S, Lawrence M, Aboyoun P, Pagès H, Gentleman R. ShortRead: A bioconductor package for input, quality assessment and exploration of high-throughput sequence data. *Bioinformatics* 2009; **25**: 2607–2608.

8 Bolger AM, Lohse M, Usadel B. Trimmomatic: A flexible trimmer for Illumina sequence data. *Bioinformatics* 2014; **30**: 2114–2120.

9 Dobin A, Davis CA, Schlesinger F, Drenkow J, Zaleski C, Jha S *et al.* STAR: Ultrafast universal RNA-seq aligner. *Bioinformatics* 2013; **29**: 15–21.

10 Pertea M, Pertea GM, Antonescu CM, Chang TC, Mendell JT, Salzberg SL. StringTie enables improved reconstruction of a transcriptome from RNA-seq reads. *Nat Biotechnol* 2015; **33**: 290–295.

11 Love MI, Huber W, Anders S. Moderated estimation of fold change and dispersion for RNA-seq data with DESeq2. *Genome Biol* 2014; **15**: 550.

12 Durinck S, Spellman PT, Birney E, Huber W. Mapping identifiers for the integration of genomic datasets with the R/ Bioconductor package biomaRt. *Nat Protoc* 2009; **4**: 1184–1191.

13 Raivo K. pheatmap: Pretty Heatmaps. R package version 1.0.12. 2019.

14 Adler D, Duncan M. rgl: 3D Visualization Using OpenGL. R package version 0.100.50. 2020.

15 Shannon P, Markiel A, Ozier O, Baliga NS, Wang JT, Ramage D *et al.* Cytoscape: A software Environment for integrated models of biomolecular interaction networks. *Genome Res* 2003; **13**: 2498–504.

16 Bindea G, Mlecnik B, Hackl H, Charoentong P, Tosolini M, Kirilovsky A *et al.* ClueGO: A Cytoscape plug-in to decipher functionally grouped gene ontology and pathway annotation networks. *Bioinformatics* 2009; **25**: 1091–1093.
