# Supplementary figures and images for: Integrated multi-omics analyses on patient-derived CRC organoids highlight altered molecular pathways in colorectal cancer progression involving PTEN
Source: J Exp Clin Cancer Res. 2021 Jun 21;40:198. doi: 10.1186/s13046-021-01986-8 (PMC8215814; doi:10.1186/s13046-021-01986-8)

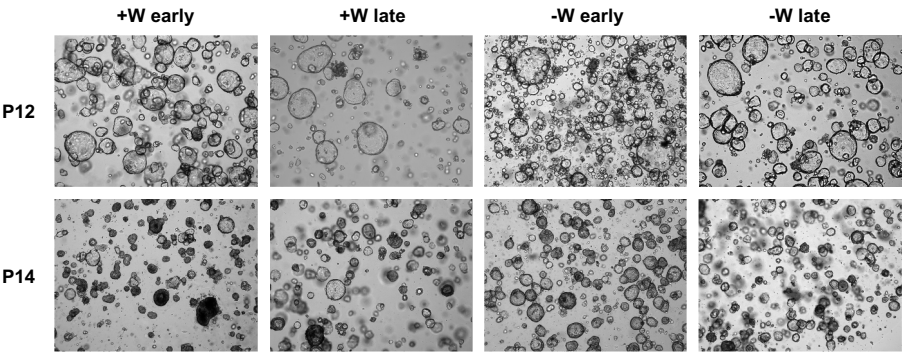

Supplement: Supplementary file 1 — Additional file 1. [file 13046_2021_1986_MOESM1_ESM.zip › Supplementary Figure 1_new.pdf]

**A**

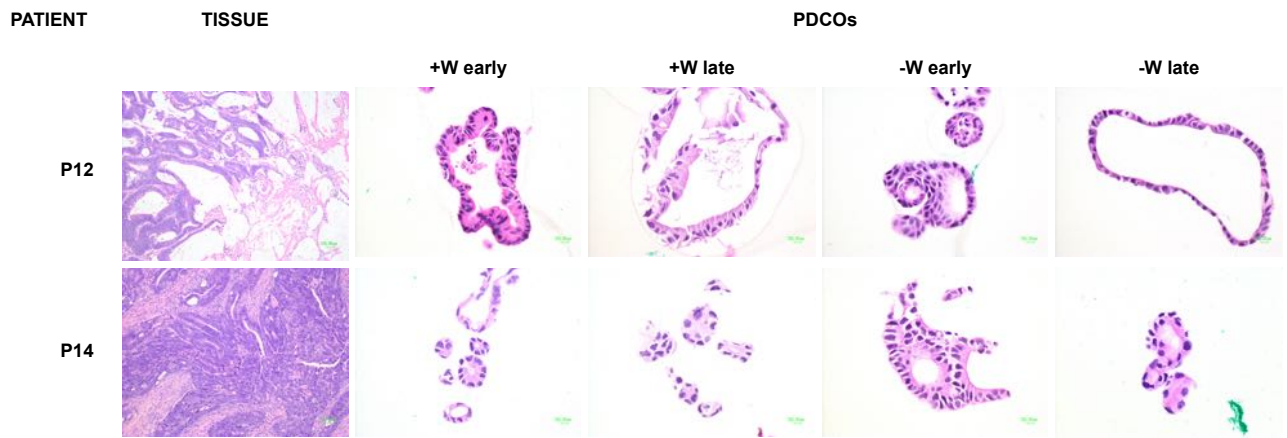

**B**

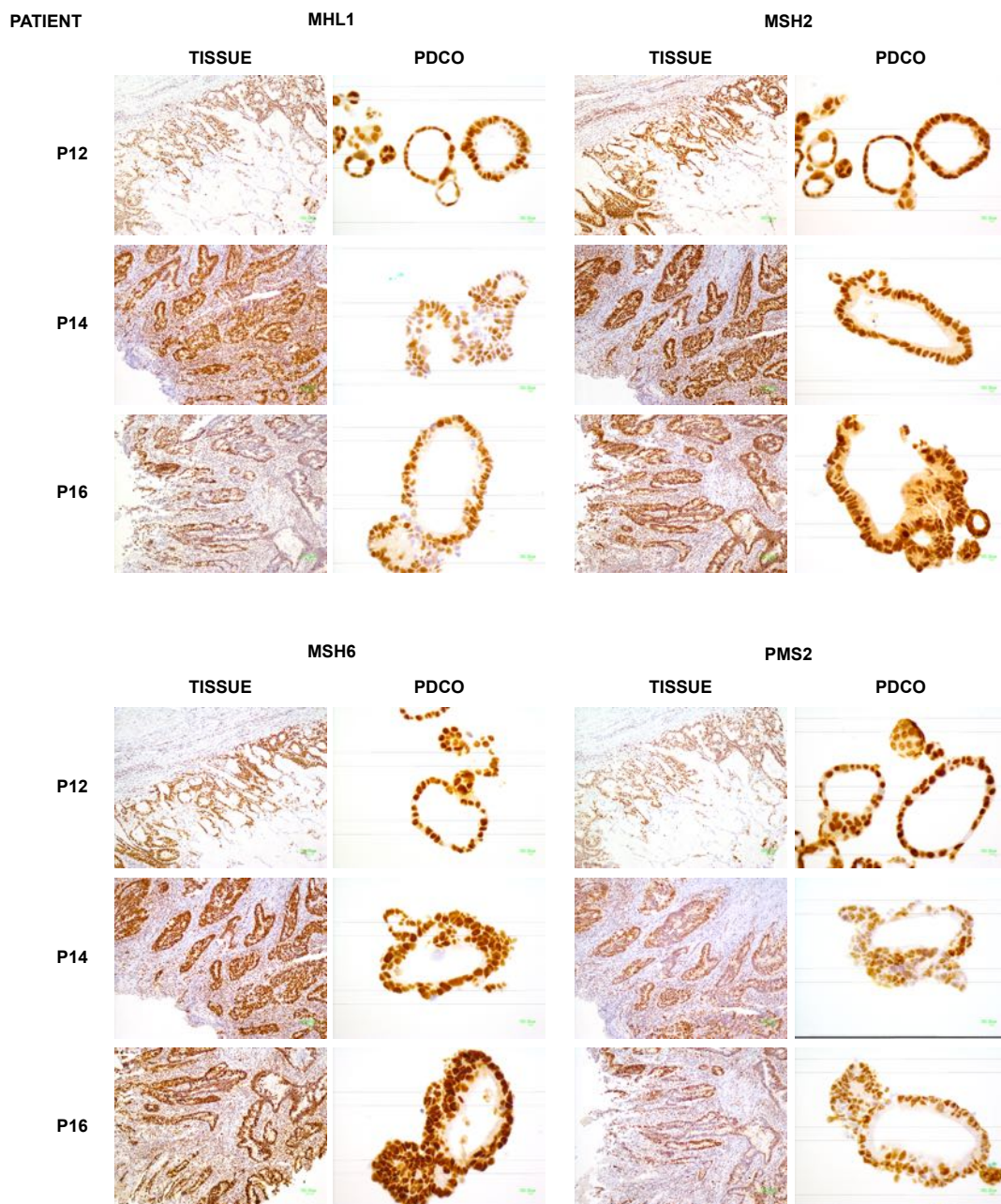

Supplement: Supplementary file 1 — Additional file 1. [file 13046_2021_1986_MOESM1_ESM.zip › Supplementary Figure 2_revised_compressed.pdf]

A

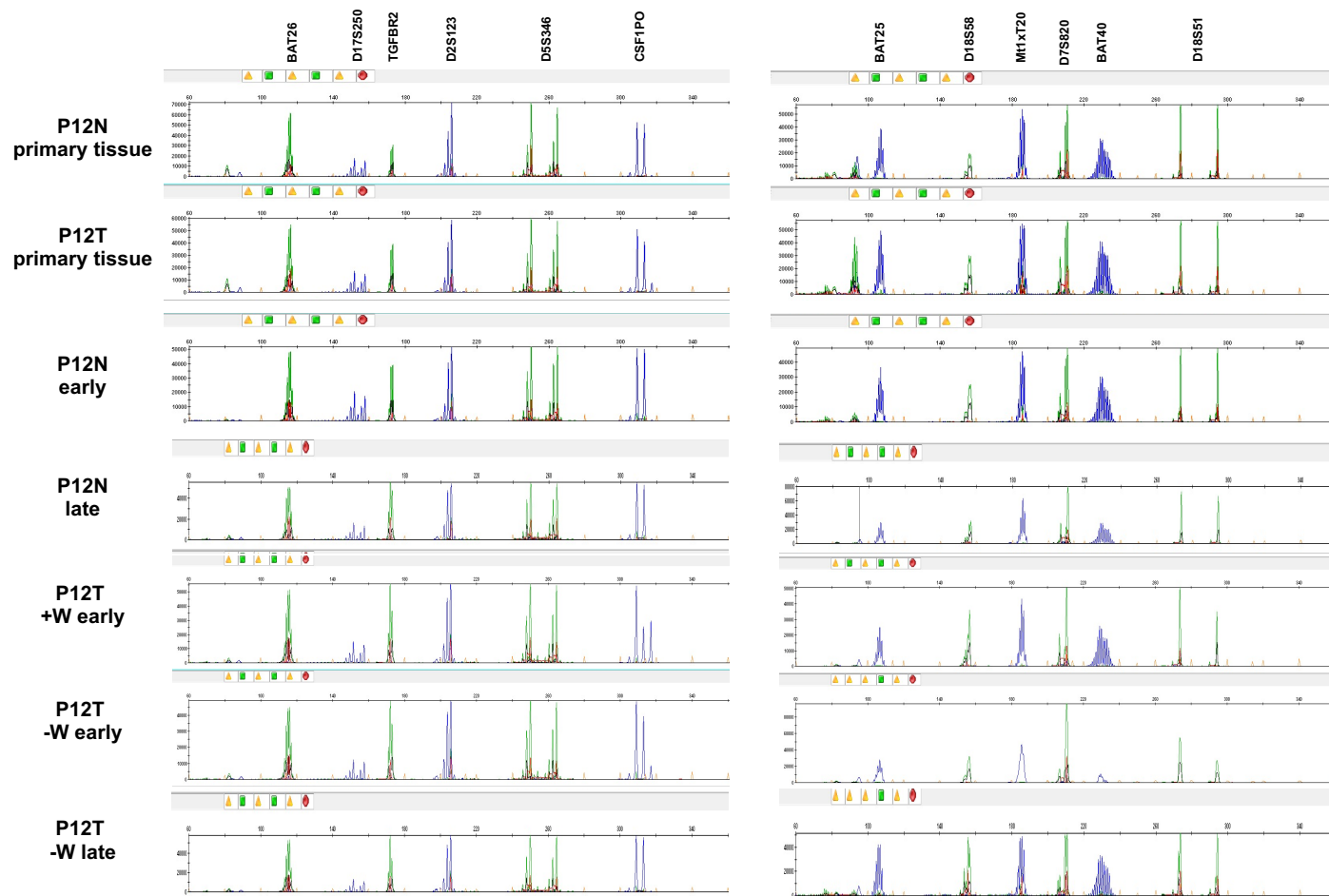

B

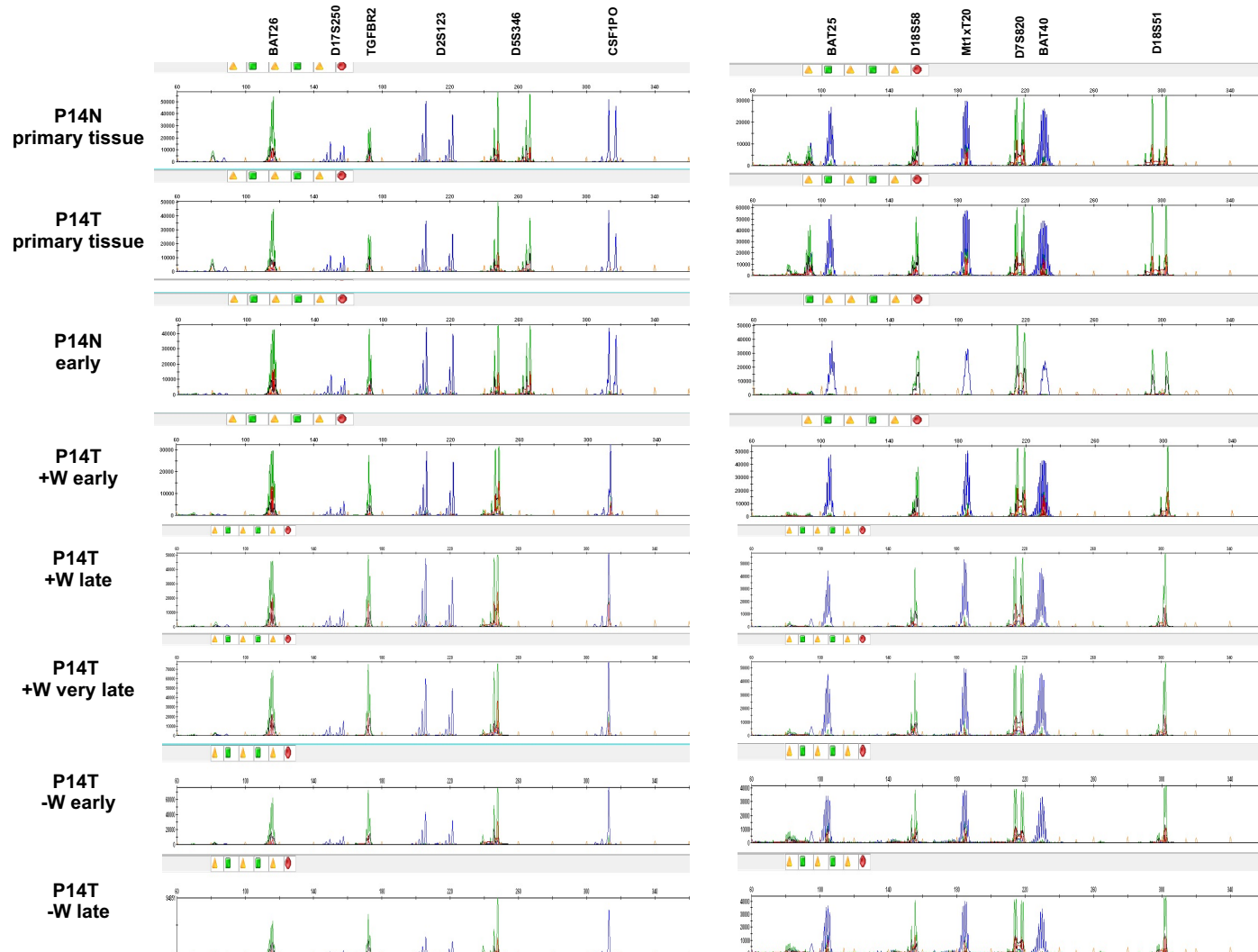

Supplement: Supplementary file 1 — Additional file 1. [file 13046_2021_1986_MOESM1_ESM.zip › Supplementary Figure 3.pdf]

Supplementary Figure 4

P14T  
-W early

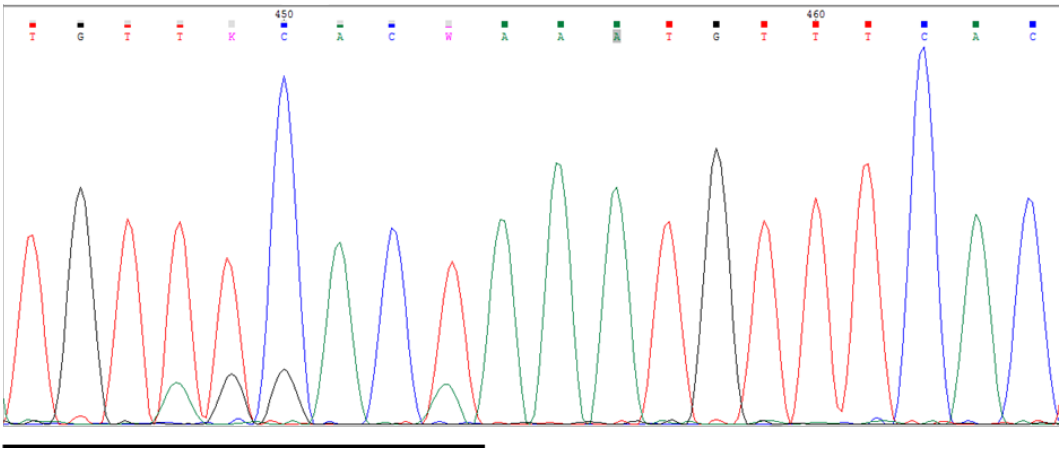

Supplement: Supplementary file 1 — Additional file 1. [file 13046_2021_1986_MOESM1_ESM.zip › Supplementary Figure 4.pdf]

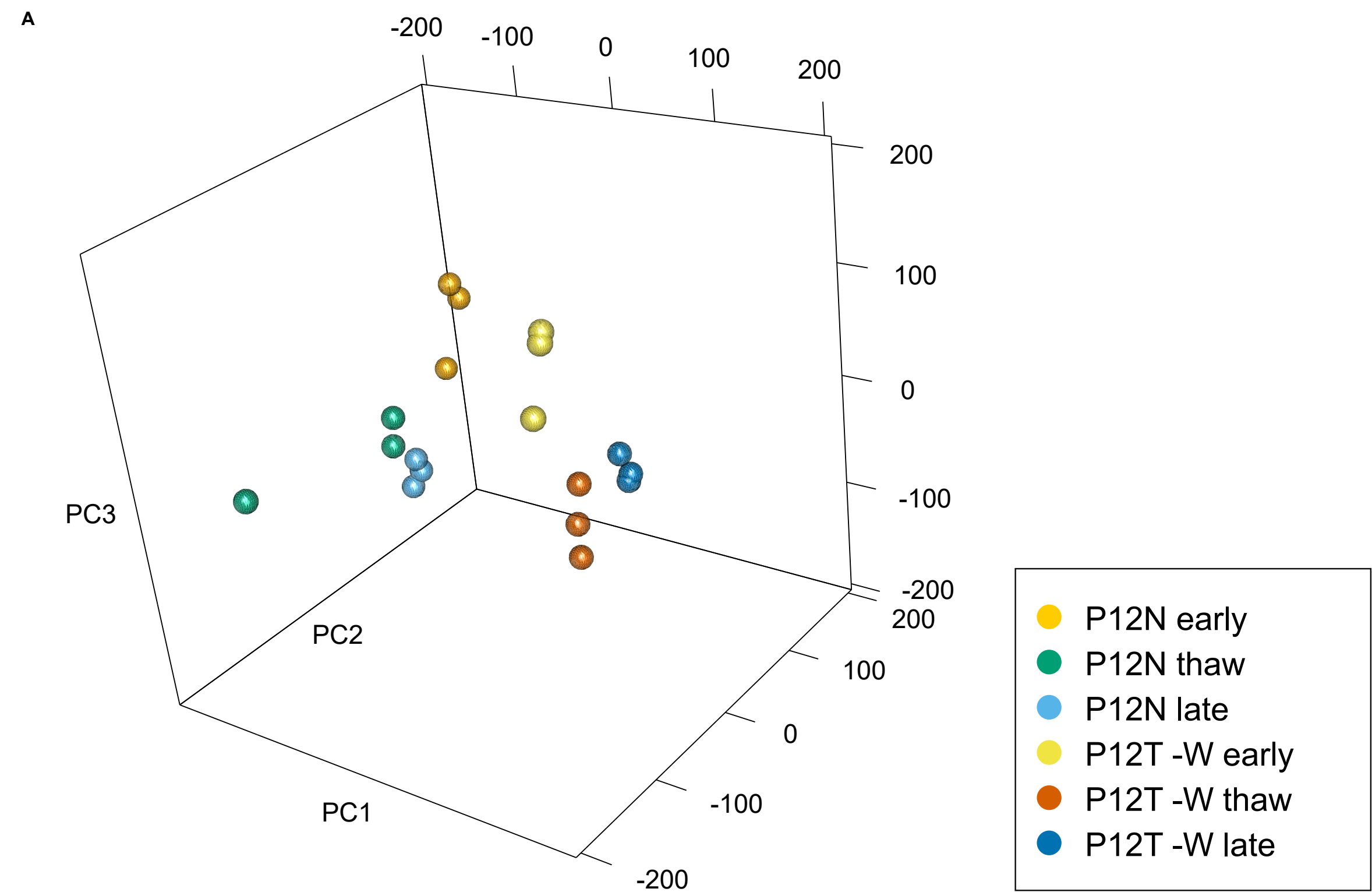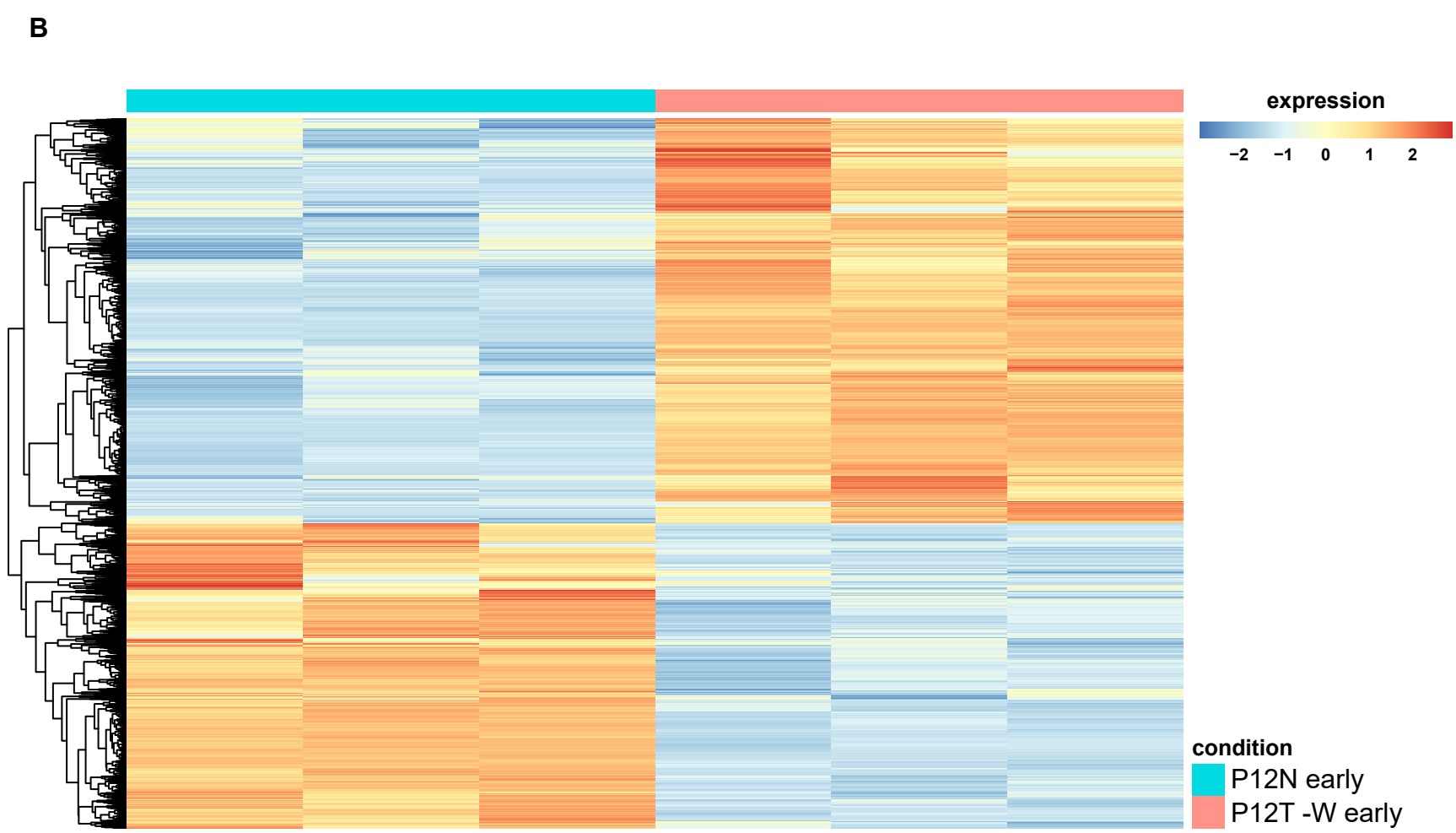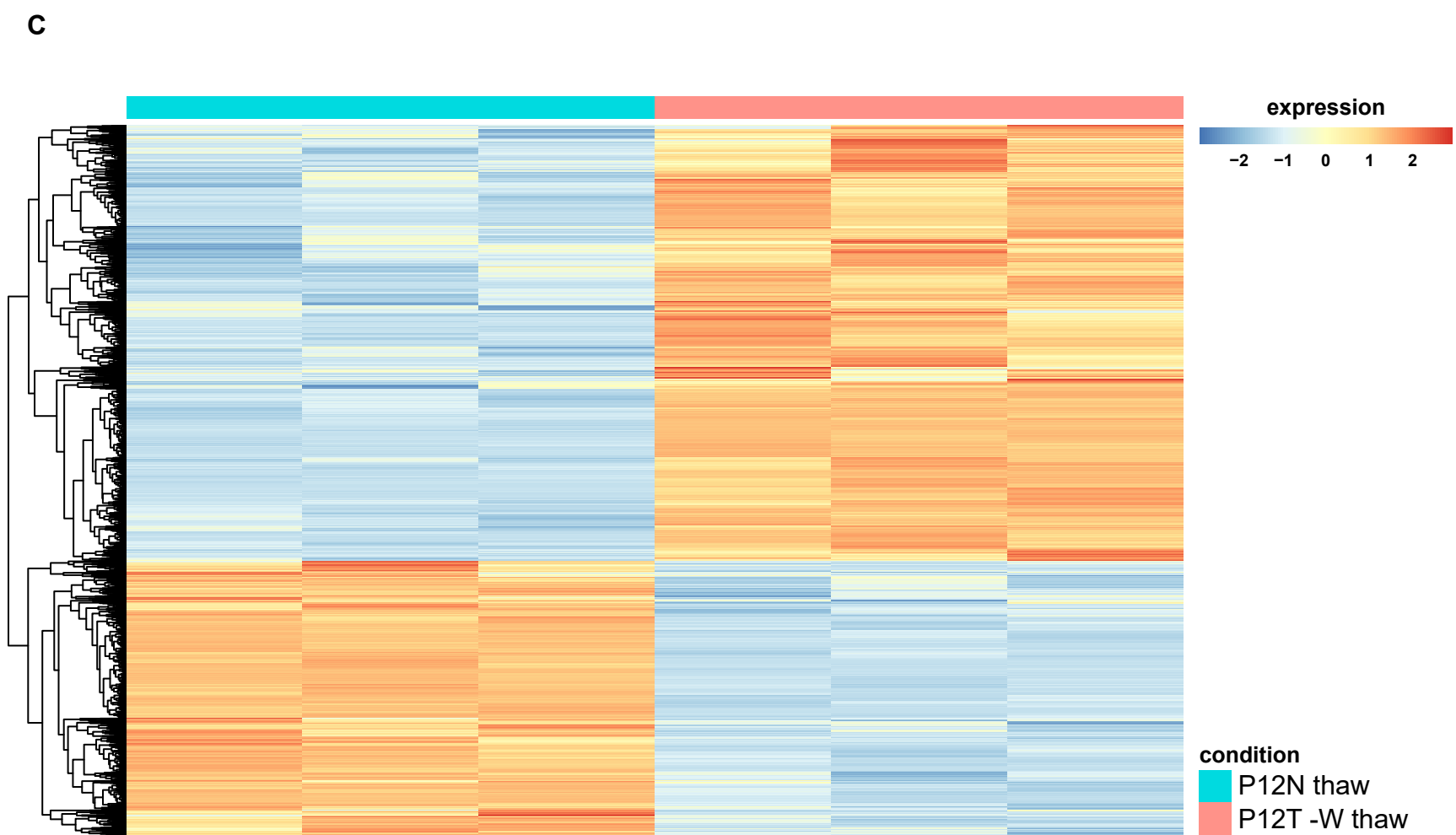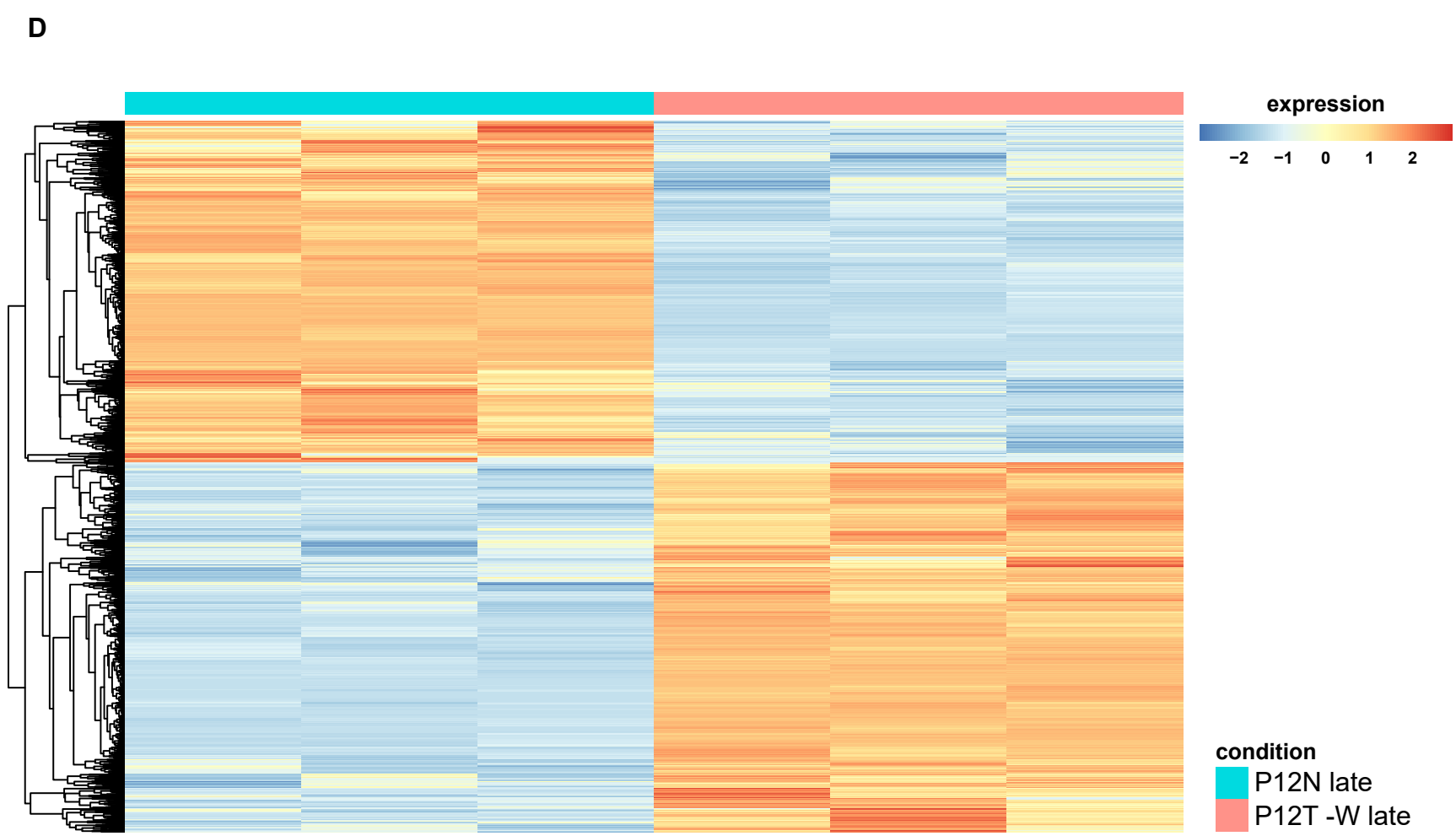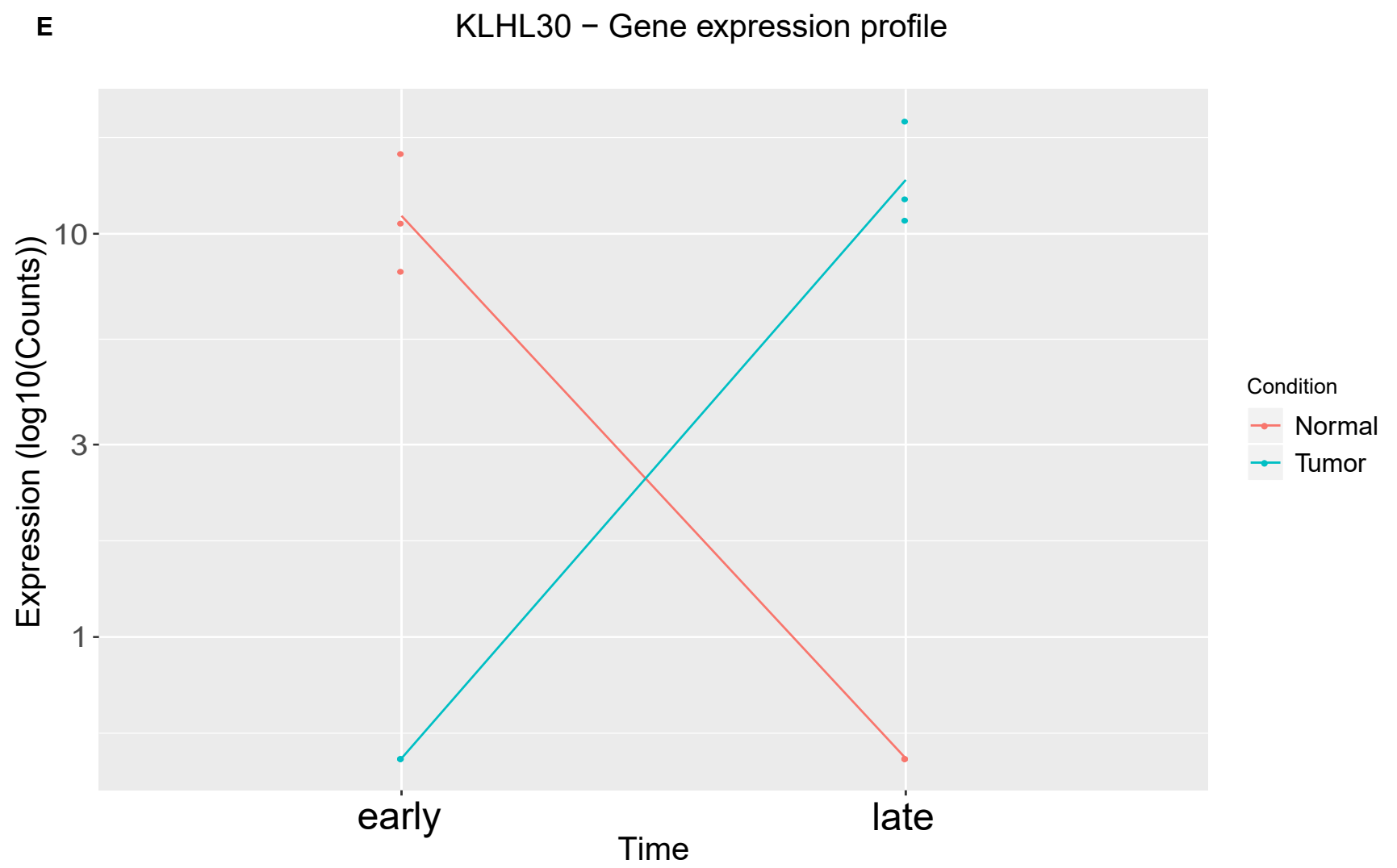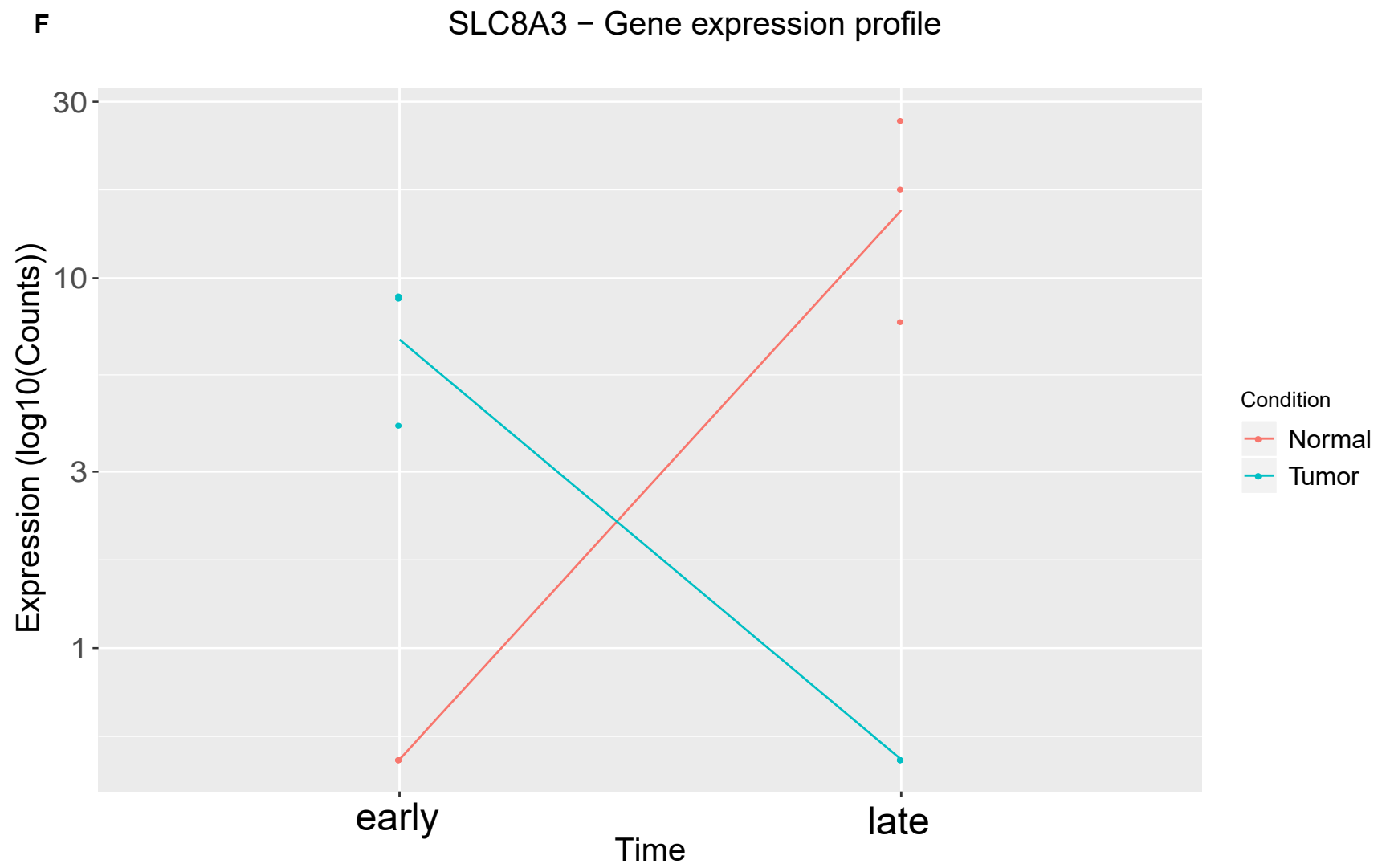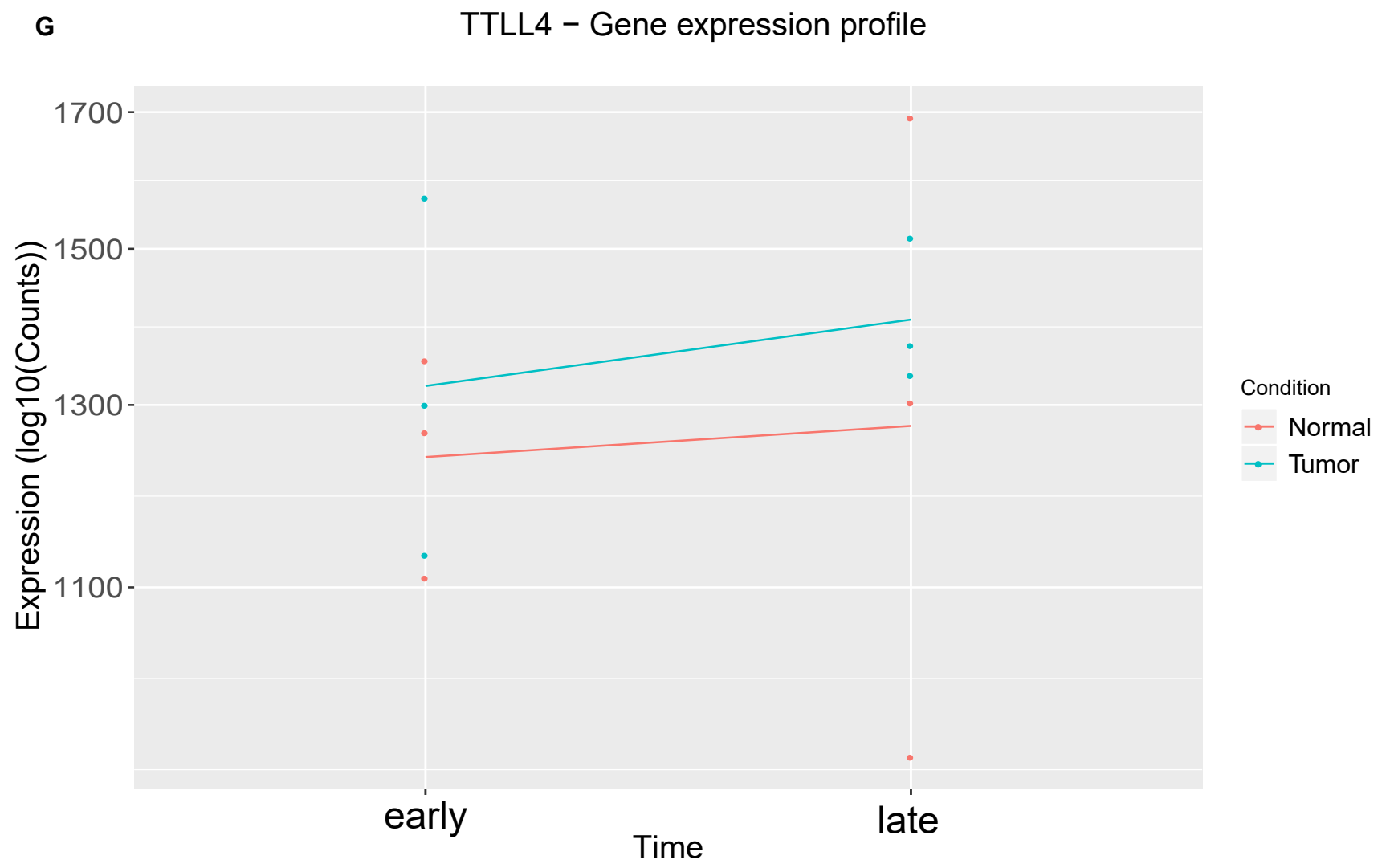

Supplement: Supplementary file 1 — Additional file 1. [file 13046_2021_1986_MOESM1_ESM.zip › Supplementary Figure 5.pdf]

Supplementary Figure 7

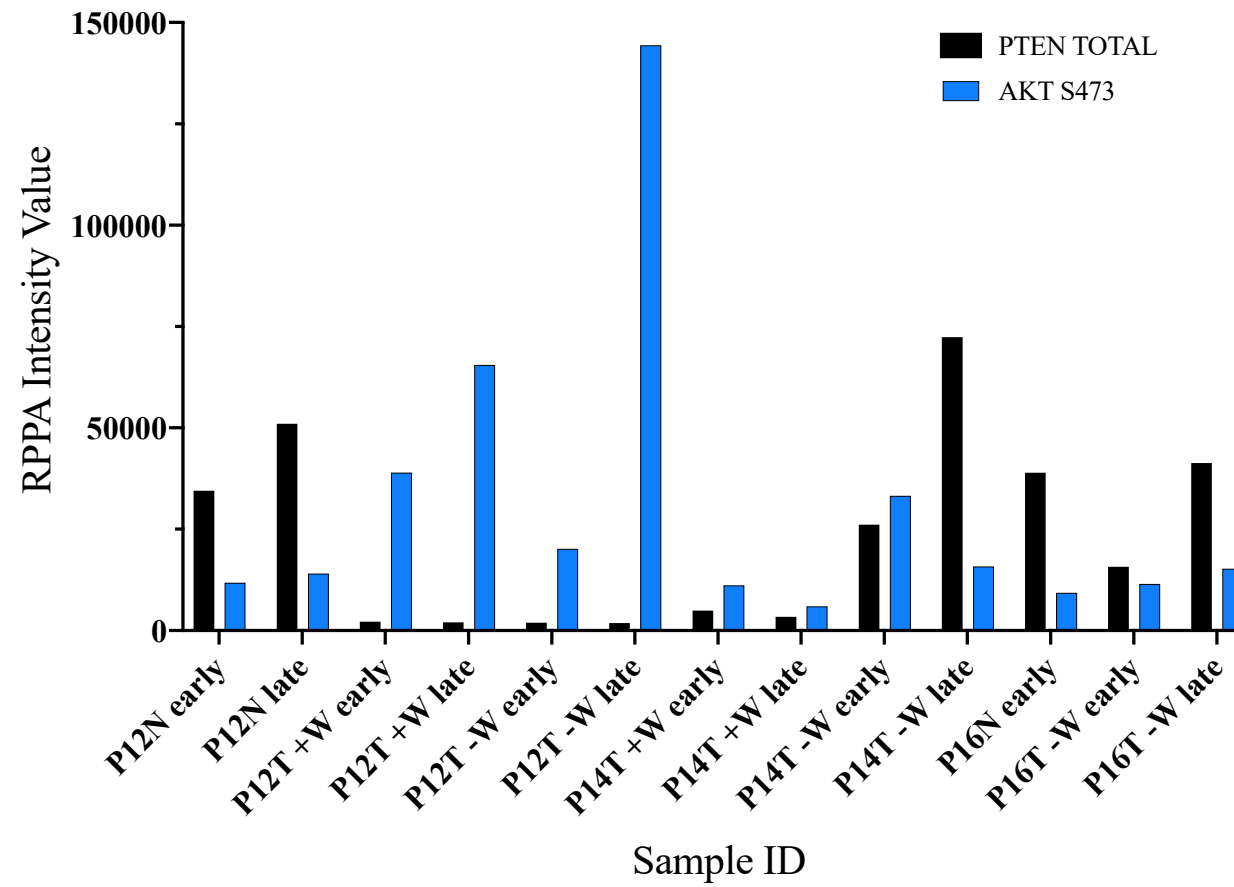

Supplement: Supplementary file 1 — Additional file 1. [file 13046_2021_1986_MOESM1_ESM.zip › Supplementary Figure 7.pdf]
